# Kinetic and Microhydrodynamic Modeling of Fenofibrate Nanosuspension Production in a Wet Stirred Media Mill

**DOI:** 10.3390/pharmaceutics13071055

**Published:** 2021-07-10

**Authors:** Gulenay Guner, Dogacan Yilmaz, Ecevit Bilgili

**Affiliations:** 1 Otto H. York Department of Chemical and Materials Engineering, New Jersey Institute of Technology, Newark, NJ 07102, USA; gg357@njit.edu; 2 Department of Mechanical and Industrial Engineering, New Jersey Institute of Technology, Newark, NJ 07102, USA; dy234@njit.edu

**Keywords:** poorly water-soluble drugs, wet stirred media milling, breakage kinetics, process modeling, microhydrodynamic model, statistical model, subset selection

## Abstract

This study examined the impact of stirrer speed and bead material loading on fenofibrate particle breakage during wet stirred media milling (WSMM) via three kinetic models and a microhydrodynamic model. Evolution of median particle size was tracked via laser diffraction during WSMM operating at 3000–4000 rpm with 35–50% (*v*/*v*) concentration of polystyrene or zirconia beads. Additional experiments were performed at the center points of the above conditions, as well as outside the range of these conditions, in order to test the predictive capability of the models. First-order, *n*th-order, and warped-time kinetic models were fitted to the data. Main effects plots helped to visualize the influence of the milling variables on the breakage kinetics and microhydrodynamic parameters. A subset selection algorithm was used along with a multiple linear regression model (MLRM) to delineate how the breakage rate constant *k* was affected by the microhydrodynamic parameters. As a comparison, a purely empirical correlation for *k* was also developed in terms of the process/bead parameters. The *n*th-order model was found to be the best model to describe the temporal evolution; nearly second-order kinetics (*n* ≅ 2) was observed. When the process was operated at a higher stirrer speed and/or higher loading with zirconia beads as opposed to polystyrene beads, the breakage occurred faster. A statistically significant (*p*-value ≤ 0.01) MLRM of three microhydrodynamic parameters explained the variation in the breakage rate constant best (R^2^ ≥ 0.99). Not only do the models and the *n*th-order kinetic–microhydrodynamic correlation enable deeper process understanding toward developing a WSMM process with reduced cycle time, but they also provide good predictive capability, while outperforming the purely empirical correlation.

## 1. Introduction

The majority of drug molecules coming out of high-throughput screening have poor water solubility due to their high lipophilicity and molecular weight [1]. Forty percent of marketed drugs are poorly water-soluble, which results in low bioavailability. There are several formulation approaches to overcome this challenge, such as amorphous solid dispersions, lipid-based formulations, and nanoparticle-based formulations [2]. Drug nanoparticles have been commonly used, as they are shown to improve the dissolution rate and bioavailability of drugs due to increased specific surface area, higher saturation solubility, and a reduction in the thickness of the diffusion layer [3,4,5]. The higher saturation solubility of nanoparticles—typically < 500 nm—has been mostly attributed to the high curvature of such particles, and explained by the Gibbs–Kelvin or Ostwald–Freundlich equations [6,7]. On the other hand, nanoparticles with sizes greater than 100 nm prepared via mechanical methods could also exhibit higher saturation solubility than bulk crystals [8], due to mechanically induced disorder and crystal defects [8,9,10,11], amorphization, and solubilization by the stabilizers [12,13]. Kesisoglou and Wu [14] estimated the solubility enhancement of 100 nm nanocrystals as compared with that of micron-sized crystals to be in the order of 10–15%, by considering a drug candidate with a molecular weight of 500 g/mol, density of 1 kg/m^3^, and a crystal medium interfacial tension of 0.015–0.020 N/m. Indeed, experimentally observed solubility enhancement via nanosizing was shown to be small: 10–15% for 100–200 nm particles [15]. Konnerth et al. [8] attributed the higher saturation solubility of 500 nm *trans*-stilbene to the mechanically induced crystal defects and disorder of the lattice structure. 

The aforementioned dissolution rate advantages led to the use of nanosuspensions and dried nanosuspensions (nanocomposites) in several marketed products with various delivery routes—including oral, parenteral, pulmonary, ocular, and dermal [13,16,17]. Nanocomposites take advantage of the high surface area and the enhanced saturation solubility of the drug nanoparticles [4,6,13]. When incorporated into solid dosage forms, they provide convenience to patients compared to drug nanosuspensions, while imparting additional physical stability to the nanoparticles during storage. In addition to the typical stabilizers—such as polymers and surfactants—that also serve as dispersants, additional dispersants may be added to the nanosuspension in order to ensure complete redispersion of nanoparticles into their primary, pre-drying state [4,13]. For example, sugars and sugar alcohols such as mannitol have been used as additional dispersants in oral formulations [13]. During drying, the sugar molecules prevent drug nanoparticles from aggregating, as water is removed and drug nanoparticles become concentrated [4]. Unfortunately, nanocomposites offer limited bioavailability enhancement for drugs with very low aqueous solubility [18], and amorphous solid dispersions (ASDs) have been preferentially used for such drugs [6]. A recent head-to-head comparison of traditional drug nanocomposites, hybrid nanocrystal ASDs (HyNASDs), and ASDs prepared by spray-drying clearly demonstrated the superior performance of ASDs, owing to their high supersaturation generation/maintenance capability [19].

In the preparation of drug nanosuspensions, wet stirred media milling (WSMM)—also known as nanosizing, nanomilling, nanonization, and wet bead milling—appeared to be the most preferred process for preparing drug nanosuspensions, according to a recent review paper that surveyed articles published between 2012 and 2017 [20]. Among the 94 studies surveyed, 45% used WSMM, 15% used high-pressure homogenization (HPH), 23% used liquid antisolvent precipitation, and the remaining 27% used a variety of top-down and bottom-up approaches. Readers are referred to the comprehensive review papers [6,20,21,22] for various methods used in the production of drug nanosuspensions. Malamatari et al. [13] presented tabular information about the nanocrystalline-based products approved by the FDA; among 17 marketed products that entailed manufacturing of drug nanosuspensions, 14 used WSMM, 2 used a bottom-up precipitation process, and only 1 used HPH [13]. All of these review papers, and the analysis of the marketed products, note that WSMM is the most widely used and preferred method for preparing drug nanosuspensions. WSMM has the capability of producing high-drug-loaded, stable nanosuspensions and it is a robust, reproducible, scalable, solvent-free, and environmentally benign process [22,23,24]. During WSMM, a pre-suspension, which contains the drug and an aqueous solution of stabilizers—i.e., polymers and/or surfactants—is circulated between a holding tank and the milling chamber, where the milling beads are retained by a screen. As the slurry is stirred by the rotor of the mill, turbulent flow emerges, causing frequent bead–bead collisions that capture, compress, and break drug particles [22]. HPH is the second most widely used top-down method [6]; it uses jet-stream homogenization by pumping the drug, dispersion medium, and stabilizers under high pressure through a micro-fluidizing nozzle. Cavitation forces, shear forces, and collision through multiple homogenization cycles result in particle size reduction [6]. A comprehensive analysis and comparison of these two processes suggested that (1) the WSMM was found to be more powerful, leading to a smaller median particle size, than the HPH; while the HPH yielded a narrower size distribution at the same median size; (2) no contaminants were observed in the HPH, regardless of the milling media used, whereas this was only possible with the use of crosslinked polystyrene in the WSMM; and (3) the HPH nanosuspensions were more physically stable at high temperatures (50–60 °C) than the WSMM suspensions [3]. Hence, HPH could offer some advantages over WSMM, depending on the intended final use and delivery route.

Despite its significant advantages, development of a WSMM poses several challenges. Most studies on WSMM have focused on formulation challenges, such as aggregation and crystal growth via Ostwald ripening (see [20,22] and references cited therein). In contrast, relatively scant information is available about the processing/operational challenges, such as energy-intensive operation, high cost due to high energy consumption, long operating hours, and contamination of drug particles by the beads [22,25,26]. Overcoming these challenges entails a mechanistic understanding of the impact of process variables—such as the stirrer speed, the size/material of the beads, and the bead loading—on the breakage kinetics, milling time required for a desired product fineness, energy consumption, and media wear [27,28]. Here, we focus on the breakage kinetics, as they relate to production cycle time: faster breakage results in shorter processing to achieve a desired nanoparticle size.

Pharmaceutical engineers/formulators and academics have used various modeling approaches to investigate the WSMM process [26]. A recent survey [26] revealed that statistics-based methods such as empirical regression and response surface methodology are the most widely used modeling approaches. On the other hand, the breakage kinetics during WSMM of drugs were also investigated using phenomenological and mechanistic models such as the population balance model (PBM) [29] and the microhydrodynamic model [27,30,31]. The effects of the bead size [24,31], stirrer speed [27,30], bead material [30], bead loading [27,30,31], and drug loading [31] on the particle size distribution of griseofulvin particles and a breakage time constant were investigated. In these studies, the use of the microhydrodynamic model shed light onto the roles of bead–bead collisions in WSMM. However, in general, these studies did not attempt to directly correlate the microhydrodynamic model parameters to the breakage rate constant, which determines the cycle time in the manufacturing of drug suspensions. Another issue is that all of these studies—and others [32,33]—used a first-order kinetics model. This model is known to have a limitation, i.e., the need to eliminate the kinetic data obtained during early milling times of WSMM [33,34]. Hence, a head-to-head fair comparison and discrimination of several kinetic models for WSMM is highly warranted—which is also within the scope of the current paper.

This study examined the impact of stirrer speed and bead material/loading on fenofibrate particle breakage during WSMM via three kinetic models and a microhydrodynamic model. Fenofibrate (FNB) was used as a challenging, model Biopharmaceutics Classification System (BCS) Class II drug (low solubility, high permeability), as it is highly prone to aggregation and Ostwald ripening [35,36]. FNB helps to reduce cholesterol and triglycerides in the blood, and is marketed as a tablet (Tricor^®^, Abbott Laboratories, Abbott Park, IL, USA), the manufacturing of which uses WSMM to prepare FNB nanosuspensions. Similarly to [37], in this study, the FNB nanosuspensions are intended to enhance the bioavailability of FNB, and they will eventually be dried into nanocomposite powders for incorporation into solid dosage forms. Experimentally, the evolution of FNB median particle size was tracked via laser diffraction during WSMM operating at 3000–4000 rpm with 35–50% (*v*/*v*) loading of polystyrene or zirconia beads in a three-factor, two-level, full factorial design of experiments (DOE). Particle sizes of FNB were measured after milling and after 7-day storage, and along with the characterization of the nanoparticles using SEM and DSC, particle change mechanisms were elucidated. In addition to the well-known first-order kinetics model, *n*th-order and warped-time kinetics models were developed; all kinetics models were fitted to the experimental data, with the objective of identifying the best kinetics model based on statistical analysis and physical plausibility considerations. The evaluation of the kinetic parameters with respect to the low and high operating conditions (stirrer speed and bead loading) as well as two different bead materials was made via the main effects plots. Main effects plots helped to visualize the influence of the milling variables on the breakage kinetics and microhydrodynamic parameters. The microhydrodynamic model parameters were calculated using the measured process variables, power consumption, and suspensions’ viscosity/density. A subset selection algorithm was used along with a multiple linear regression model (MLRM) to delineate how the breakage rate constant *k* was affected by the microhydrodynamic parameters. As a comparison, a purely empirical correlation for *k* in terms of the process parameters and bead properties was also developed. Four additional WSMM experiments were conducted at the center points and outside the domain of the DOE to test the predictive capability of the kinetic–microhydrodynamic correlation and the purely empirical correlation. The limitations of the models, as well as future strategies for the development and improvement of predictive models, were discussed. Overall, this study offers the first comprehensive treatment of breakage kinetics during WSMM in view of the fundamental physics (microhydrodynamics), and explores the microhydrodynamic parameters that govern the breakage kinetics. It is expected that such a comprehensive analysis of breakage kinetics with consideration of the actual physics in the mill will provide insights, quantitative understanding of the process regarding the impact of process parameters/bead material on the breakage kinetics and cycle time, and some predictive capability.

## 2. Materials and Methods

### 2.1. Materials

Fenofibrate (FNB, BP grade)—which is a BCS Class II drug with an aqueous solubility of 0.8 mg/L at room temperature [38]—was purchased from Jai Radhe Sales (Ahmedabad, India). The suspension formulation included a nonionic cellulosic polymer (HPC: hydroxypropyl cellulose, L grade, Nisso America Inc, New York, NY, USA) and an anionic surfactant (SDS: sodium dodecyl sulfate, ACS grade, GFS chemicals, Columbus, OH, USA). Zirmil Y grade YSZ beads with a density of 6000 kg/m^3^ were purchased from Saint-Gobain ZirPro (Mountainside, NJ, USA). CPS beads with a density of 1040 kg/m^3^ were purchased from Norstone Inc. (HCC grade, Bridgeport, PA, USA). While the nominal sizes of both beads are 400 µm, the actual median sizes of CPS and YSZ beads were measured as 444 µm and 405 µm, respectively, via laser diffraction using a HELOS/RODOS particle size analyzer (Sympatec, NJ, USA) in dry dispersion mode. Microhydrodynamic calculations were performed using the actual median sizes of the beads. Drug suspensions were prepared in deionized water as the liquid medium.

### 2.2. Methods

#### 2.2.1. Wet Stirred Media Milling

The formulation was selected according to the detailed stability studies by our group on FNB [35,36,39]. We prepared 235 g pre-suspensions with 10% FNB, 7.5% HPC-L, and 0.05% SDS with respect to 200 g DI water under constant shear mixing (Cat#. 14-503, Fisher Scientific, Pittsburgh, PA, USA) for 2 h at 300 rpm. As will be demonstrated in Section 3.1, not only did this particular formulation ensure physical stability and mitigate the aggregation and growth of FNB particles via ripening, but also it built up sufficiently high suspension viscosity, which in turn led to an accurate measurement of the power consumption during the milling and accurate calculation of the microhydrodynamic parameters. After overnight storage at 8 °C, the pre-suspensions were milled using a MicroCer wet stirred media mill (Netzsch Fine Particle Size Technology, LLC, Exton, PA, USA) for 180 min using the process variables presented in Table 1. Runs 1–8 correspond to a 3-factor, 2-level, full factorial DOE. The experimental data from these runs were used in the kinetic parameter estimation. The low–high values of the stirrer speed and the bead loading, as well as the total milling time for the DOE, were selected based on our prior wet milling studies using FNB [35,36], the limitations of our equipment, and the requirements of the kinetic models (refer to Section 2.2.3). The design limit of the equipment (4200 rpm) dictates 4000 rpm as the high value with a safety margin, while bead loading above 0.50 would entail more frequent, additional intermittent cooling; hence, it was not selected. Runs 9 and 10, corresponding to the center points of the DOE for CPS and YSZ, as well as Runs 11 and 12, whose variables were selected outside the domain of the DOE, were used to test the predictive capability of the *n*th-order kinetic-model–microhydrodynamic-model correlation (i.e., *k* = *k*(*a*, *σ*_b_^max^, *α*_b_, *Πσ*_y_)) and the purely empirical correlation (i.e., *k* = *k*(*ω*, *c*, *ρ*_b_, *Y*_b_)). As *ω* was close to the design limit, we could not increase it beyond the high value (4000 rpm) of the DOE; hence, for Runs 11 and 12, *ω* and *c* were both selected to be 15% smaller than the respective low values in the DOE (Runs 1 and 2). As will be demonstrated later, Runs 11 and 12 were quite different from Runs 1–8 in terms of the observed breakage kinetics. Practically, such low energetic conditions, inducing relatively low power consumption and heat dissipation, may be used to handle temperature-sensitive drugs, minimize the extent of amorphization and form conversion [4], and reduce YSZ bead contamination [24]. As we expected a slower breakage in Runs 11 and 12, based on the kinetic results from Runs 1–8, we purposefully prolonged the milling to 7 h for CPS (Run 11) and 6 h for YSZ (Run 12), thus attempting to meet the requirements in Section 2.2.3.

The mill had a chamber volume *V*_mc_ of 80 mL, lined with zirconia, and a zirconia shaft. The bead loadings were calculated by dividing the true volumes of the beads by the volume of the milling chamber (*v*/*v*). Suspensions were recirculated between the holding tank and the milling chamber at a volumetric flow rate of 126 mL/min, using a peristaltic pump (Cole-Palmer, Master Flex, Vermont Hills, IL, USA). A 200-µm nominal-sized stainless-steel screen was used to hold the beads in the milling chamber. The temperature of the milling chamber and the holding tank was kept below 35 °C using a chiller (Model M1-.25A-11HFX, Advantage Engineering, Greenwood, IN, USA). Due to the limited cooling capacity of our chiller, YSZ beads caused overheating, especially at the high stirrer speed; hence, intermittent cooling was applied, as in [24,30,40], to keep the temperature below 35 °C. Samples were taken from the outlet of the mill at certain time intervals (2*^s^*, *s* = 0, 1, 2,...7), with additional time points of 40 s, 24 min, 48 min, 96 min, and 180 min. The sampling procedure was modified to accommodate longer milling in Runs 11 and 12. The final sample was taken from the holding tank, and all samples were characterized.

#### 2.2.2. Characterization Techniques 

The particle-size distribution (PSD) of the FNB suspensions at various milling times was determined by laser diffraction using an LS 13-320 Beckman Coulter instrument (Brea, CA, USA). Polarized intensity differential scattering (PIDS) was kept between 40% and 50%, while the obscuration was maintained below 8% in all measurements. The software computed the PSD using the Mie scattering theory by taking the refractive indices of FNB and the measurement medium (water) (1.55 and 1.33, respectively). Prior to each measurement, about 1.0 mL of suspension sample was dispersed into 5.0 mL of the stabilizer solution of the used formulation, using a vortex mixer (Fisher Scientific Digital Vortex Mixer, Model No: 945415, Pittsburgh, PA, USA) at 1500 rpm for 1 minute. Measurements were repeated four times (*n* = 4), and the average and standard deviation (SD) of these measurements were calculated. To assess the physical stability after 7 days and assess aggregation/Ostwald ripening upon ageing, the milled nanosuspensions were stored at 8 °C in the refrigerator. They were mixed for 30 min prior to the particle size measurement and allowed to reach room temperature.

The apparent shear viscosities *µ*_L_ of the milled suspensions were measured using an R/S plus rheometer (Brookfield Engineering, Middleboro, MS, USA) with a Lauda Eco water jacket assembly (Lauda-Brinkmann LP, Delran, NJ, USA). A CC40 coaxial cylinder with a jacketed setup was used to impart a controlled shear rate on the samples from 0 to 1000 1/s for 60 s at 25 ± 0.5 °C. The raw data were analyzed using the Rheo3000 software, and the apparent shear viscosity at 1000 1/s was used in the microhydrodynamic model. The density was calculated in triplicate based on the weight of 35 mL of nanosuspension.

Run 10 was selected for solid-state characterization, since it was milled using the center point of the DOE with the YSZ beads. The nanosuspension was poured into a petri dish as a thin layer and allowed to dry overnight in a vacuum chamber. Approximately 6–7 mg samples of the milled and dried particles, the physical mixture, the as-received fenofibrate, and the HPC-L were weighed, put in a 40 µL aluminum pan, and sealed. A Mettler Toledo polymer analyzer DSC (Model DSC 3, Columbus, OH, USA) was used to determine the fusion enthalpy and the melting point of the FNB. All of the samples were heated at a rate of 5 °C/min, with a temperature range of 25–150 °C. Nitrogen was used at a flow rate of 60 mL/min. Data analysis was performed using the STARe V16.20 software provided by Mettler Toledo (Columbus, OH, USA).

Before scanning electron microscopy (SEM), 0.1 mL of the Run 10 nanosuspension was diluted with 10 mL deionized water and centrifuged (Compact II centrifuge, Clay Adams^®^ Brand, Sparks, MD, USA) at 3200 rpm for 10 min to separate the drug from the aqueous phase with excess polymer. This dilution–centrifugation process was repeated two more times, where 8 mL of the aliquot was decanted and replaced with fresh deionized water. After the third step, a droplet from the aliquot of the sample was put on top of a carbon specimen holder, and then placed in a desiccator for overnight drying under vacuum. The dried sample was then sputter-coated with gold using a BAL-TEC MED020 (BAL-TEC, Balzers, Switzerland) to reduce possible charging during SEM imaging. The morphology of the particles was examined using a JEOL JSM 7900F field emission SEM (JEOL USA, Inc., Peabody, MA, USA) operated at 2 kV. SEM images were taken at ×15 k and ×30 k magnification. 

#### 2.2.3. Kinetic Models 

In the preparation of drug nanosuspensions via WSMM, breakage of the particles in a well-stabilized suspension is expected to be the dominant mechanism as compared to aggregation and particle growth (due to ripening) [41]. Therefore, the median particle size *d*_50_ monotonically decreases over time *t* until a limiting size *d*_lim_—also known as the grinding limit—is attained or approached during prolonged milling [36]. Two experimental requirements emerge for an accurate and meaningful kinetic analysis: (1) the experimental *d*_50_–*t* profile should attain or approach an asymptote (limiting size) at sufficiently long, yet practically feasible, milling times; and (2) it should not exhibit a size increase, regardless of the underlying mechanism. As will be demonstrated in Section 3, these requirements were largely met, due to judicious selection of the formulation and process conditions. When these requirements are met, the breakage rate may be described by a breakage rate constant *k* and the difference between the median particle size and the limiting size raised to the power *n*:

(1) $$dd_{50}\left(t\right)/dt=-k{\left[d_{50}\left(t\right)-d_{\lim}\right]}^{n}\;\;\mathrm{with}\;\;\;d_{50}\left(0\right)=d_{50,0}$$

In previous investigations, *n* was commonly taken as 1 (first-order kinetics), and application of separation of variables followed by integration of both sides yielded the following widely used model [30,31,42,43]:

(2) $$d_{50}\left(t\right)=d_{\lim}+\left(d_{50,\;0}-d_{\lim}\right)\exp\left(-kt\right)\;\;\;\;$$

Here, *d*_50,0_ is the initial median size. Equation (2) with a single process time constant *τ*_p_ = 1/*k* was not able to fit all of the experimental data governed by two or potentially more characteristic time constants [44]. Hence, in many studies (e.g., [33,34]), the initial median particle size at the 0th min was discarded, thus making the first timepoint (e.g., 1st min median size) the initial size for better fitting capability. Depending on the initial PSD and initial breakage kinetics, even eliminating one data point may not allow for the accurate fitting of the experimental data. Nevertheless, eliminating data points is reflective of poor robustness of the model, and was not practiced in this study.

As a commonly used approach in chemically reacting systems [45,46], an *n*th-order kinetics model may be considered with the objective of resolving the inadequacy of the first-order kinetics model. Separation of the variables in Equation (1) with *n* ≠ 1, followed by integration of both sides, yielded the following equation:

(3) $$d_{50}\left(t\right)=d_{\lim}+{\left[{\left(d_{50,0}-d_{\lim}\right)}^{1-n}-\left(1-n\right)kt\right]}^{1/\left(1-n\right)}$$

To the best of the authors’ knowledge, this general *n*th-order model has not been used previously for describing the breakage kinetics in WSMM. Note that in [46] a second-order kinetics model was assumed, without fitting *n*, to describe the mass fraction of only coarse particles as a function of time—not the median size of the whole PSD—and was only applied to the dry ball milling of narrow, coarse sieve cuts > 2.5 mm; this model did not consider the grinding limit or *d*_lim_ either. 

Another kinetic modeling approach entails consideration of a time-dependent breakage rate parameter *k*(*t*), as follows:

(4) $$dd_{50}\left(t\right)/dt=-k\left(t\right)\left[d_{50}\left(t\right)-d_{\lim}\right]\;\;\mathrm{with}\;\;\;d_{50}\left(0\right)=d_{50,0}$$

with *k*(t) = *k*_0_*κ*(t). Unlike the constant *k* in Equations (1–3), here, *k*_0_ is a breakage rate constant. The time-dependent function *κ*(t) can be obtained from a first-order differential equation dϕ/d*t* = *κ*(*t*) with ϕ(0) = 0 at *t* = 0, where ϕ is referred to as the warped (false) time. This warped-time concept was previously introduced in the context of a population balance model [47,48], but it has not been used to derive the evolution of median size, as done here for the first time. With the above defining differential equation for ϕ, Equation (4) was transformed to a first-order differential equation in ϕ, as follows:

(5) $$dd_{50}\left(\phi \right)/d\phi =-k_{0}\left[d_{50}\left(\phi \right)-d_{\lim}\right]\;\;\;\mathrm{with}\;\;\;d_{50}\left(0\right)=d_{50,0}$$

We take $\phi =t^{n}$ without loss of generality, as it is the simplest relation with one parameter (*n*) that satisfies the initial condition for ϕ, and simply reduces to the first-order model when *n* = 1. Hence, the breakage rate parameter takes the form:$\;k\left(t\right)=k_{0}nt^{n-1},$ and the solution of Equation (5) leads to the following warped-time kinetic model:

(6) $$d_{50}\left(t\right)=d_{\lim}+\left(d_{50,0}-d_{\lim}\right)\exp\left(-k_{0}t^{n}\right)$$

The Levenberg–Marquardt algorithm was used to fit the three models described by Equations (2), (3), and (6) to the logarithm of the experimental median sizes, and *d*_lim_, *k* or *k*_0_, and *n* were estimated using SigmaPlot software (Version 11, Systat Software, Inc., San Jose, CA, USA). In the fitting, *d*_lim_ was constrained to be smaller than the final median particle size [30]. Statistical analysis of the fits was performed to discriminate the models, and main effects plots of the parameters were prepared.

#### 2.2.4. Microhydrodynamic Analysis

A microhydrodynamic model was developed by Eskin et al. [49,50] to determine the mean velocity of bead oscillations in well-mixed slurries, using the kinetic theory of granular flows and fundamental granular energy balance [51]; Bilgili and Afolabi [34] modified this model and applied it to an actual WSMM process. For brevity, only essential equations are recorded here, and readers are referred to [34,49,50] for all details of the assumptions and derivation steps. Via three different mechanisms, the power applied per unit volume of slurry *P*_w_ inside a wet stirred media mill dissipates as follows:

(7) $$P_{w}=\varepsilon_{\mathrm{visc}}+\varepsilon_{\mathrm{coll}}+\varepsilon_{\mathrm{ht}}$$

where *ε*_visc_ is the energy dissipation rate due to both the liquid–beads viscous friction and lubrication, *ε*_coll_ is the energy dissipation rate due to partially inelastic bead–bead collisions, and *ε*_ht_ is the power spent on shearing the slurry at the same shear rate without the beads [34]. A major assumption is that the power applied by the mill stirrer is uniformly applied throughout the whole volume of the slurry, and equals the total energy dissipation rate *ε*_tot_ [49]. It is assumed that the flow in the milling chamber is in the developed turbulence regime, and that the turbulence intensity is high enough to overcome the radial stratification so that the beads are uniformly dispersed in the milling chamber. The fluctuating velocities of the beads are assumed to follow a Maxwellian distribution. The Hertz theory for the nearly elastic impact of beads is used. If the (drug) particle loading is low (<10% *v*/*v*) and the (drug) particles are much smaller than the beads, the collision of two beads is considered to be the interaction between two elastic spheres. In addition to these assumptions, timewise variation of the power consumption and granular temperature was not considered; the timewise averaged power consumption and the density and viscosity of the final milled suspension were used in the calculations. Finally, in the calculation of bead- and drug-particle-dependent quantities, the median size was assumed to represent the population; hence, the potential impact of the polydispersity was not considered. Other assumptions can be found in [49]. The three dissipation rates in Equation (7) can be expressed as follows:

(8) $$P_{w}=\frac{54\mu_{L}c\theta R_{\mathrm{diss}}}{d_{b}^{2}}+\frac{12}{d_{b}\sqrt{\pi }}\left(1-e^{2}\right)g_{0}c^{2}\rho_{b}\theta^{3/2}+\varepsilon_{\mathrm{ht}}$$

where *µ*_L_ is the apparent shear viscosity of the milled suspension, *c* is the bead loading (fractional volumetric concentration), *θ* is the granular temperature defined as the bead-milled suspension’s relative mean square velocity, *R*_diss_ is the effective dissipation coefficient, *d*_b_ is the median size of the beads, *e* is the restitution coefficient for the bead–bead collisions (0.9 and 0.76 for CPS and YSZ beads, respectively) [52,53], *ρ*_b_ is the density of the beads, and *g*_0_ is the radial distribution function. Unlike [49,50], we used a more accurate *g*_0_ model here, i.e., the Lun model [54]. As the bead loadings are high, the bead volume fraction at random packing *c*_lim_ = 0.63 [55] must be factored in for the *g*_0_ calculation, as follows:

(9) $$g_{0}={\left[1-{\left(c/c_{\lim}\right)}^{1/3}\right]}^{-1}$$

We measured *µ*_L_, *ρ*_L_, and *P*_w_ (reported in Table S1 of the Supplementary Materials); *ε*_ht_ was found to be negligibly small and disregarded, similar to [30]. The fsolve function in MATLAB (see Section S1 in the Supplementary Materials for a sample MATLAB code) was used to solve for the only unknown in Equation (8)—i.e., *θ*—which was then used to calculate the frequency of single-bead oscillations *ν* and the average oscillation velocity of the beads *u*_b_, as follows:

(10) $$\nu =\frac{24c}{d_{b}}g_{0}\sqrt{\frac{\theta }{\pi }}$$

(11) $$u_{b}={\left(8\theta /\pi \right)}^{1/2}$$

The beads capture the drug particles and compress them. The maximum contact pressure at the center of the contact circle *σ*_b_^max^ of the two colliding beads—which was derived by considering the elastic contact deformation of the beads along with the elastic/perfectly plastic deformation of the particles caught between the beads—was calculated as:

(12) $$\sigma_{b}^{\max}=\frac{3F_{b}^{n}}{2\pi \alpha_{b}^{2}}$$

where *F*_b_^n^ (refer to Appendix A) and *α*_b_ are the average maximum normal force during the collision of two elastic beads, and the radius of the contact circle formed at the contact of two beads, respectively. *α*_b_ was determined by the following equation:

(13) $$\alpha_{b}={\left[\frac{3\left(1-\eta_{b}^{2}\right)}{4Y_{b}}R_{b}F_{b}^{n}\right]}^{1/3}$$

where *η*_b_ and *Y*_b_ are Poisson’s ratio and Young’s modulus of the bead material, respectively, and *R*_b_ is the radius of the beads. The values of *η*_b_ and *Y*_b_ were taken as 0.33 and 1.5 GPa for CPS beads [56], and 0.2 and 200 GPa for YSZ beads [57], respectively. The multiplication of the probability *p* of a single drug particle caught between beads (refer to Equation (A7)) and the frequency of single-bead oscillations *ν* (Equation (10)) gives the average frequency of drug particle compressions *a*:

(14) $$a=p\nu =11.64\frac{c^{2}g_{0}}{\sqrt{\pi \left(1-c\right)}}{\left[\frac{\rho_{b}\left(1-\eta_{b}^{2}\right)}{Y_{b}}\right]}^{2/5}\frac{R_{p}}{R_{b}^{2}}\theta^{9/10}$$

where *R*_p_ is the initial radius of the drug particles. The energy dissipation rate resulting from the deformation of the particles per unit volume *Π* is determined as follows:

(15) $$\Pi =4.46\frac{c^{2}g_{0}}{\pi^{5/2}{\Psi \sigma }_{y}}{\left(\frac{Y_{b}}{1-\eta_{b}^{2}}\right)}^{18/15}{\left(\frac{Y^{*}}{Y_{p}}\right)}^{\gamma }\rho_{b}^{4/5}\frac{R_{p}}{R_{b}^{2}}\theta^{13/10}$$

In Equation (15), Ψ, *σ*_y_, Y*, and *γ* are the volume fraction of the drug particles in the suspension (0.074), contact pressure in a drug particle captured when the fully plastic condition is reached, reduced Young’s modulus of the bead–drug-particle contact, and *γ* a coefficient that can be taken as 1/3 for elastic contact between the particle and the bead [49], respectively. Young’s modulus and Poisson’s ratio for the FNB particles were taken as 8.93 GPa and 0.3, respectively [58]. Since a reliable value for *σ*_y_ of the FNB particles was not found in the literature, a pseudo energy dissipation rate, similar to [30], was calculated as follows:

(16) $$\Pi \sigma_{y}=4.46\frac{c^{2}g_{0}}{\pi^{5/2}\Psi }{\left(\frac{Y_{b}}{1-\eta_{b}^{2}}\right)}^{18/15}{\left(\frac{Y^{*}}{Y_{p}}\right)}^{\gamma }\rho_{b}^{4/5}\frac{R_{p}}{R_{b}^{2}}\theta^{13/10}$$

#### 2.2.5. Multiple Linear Regression and Subset Selection Algorithm

Main effects plots were drawn to visualize how the microhydrodynamic parameters varied upon changes in the stirrer speed and loading of the beads with different materials (CPS and YSZ) in the 3-factor, 2-level DOE. In addition, a relationship between the calculated microhydrodynamic parameters and the breakage rate constant *k* of the *n*th-order kinetics model of the general form *k* = *k* (*a*, *σ*_b_^max^, *α*_b_, *Πσ*_y_) was sought by the subset selection algorithm (see Algorithm 1 below), which was modified from [59]. Our analysis includes three multiple linear regression model (MLRM) approaches to determine the relationship between the breakage rate constant *k* (response) and four microhydrodynamic parameters *σ*_b_^max^, *α_b_*, *a*, and *Πσ_y_*, i.e., first-order MLRM, second-order MLRM, and MLRM with interaction terms. For all three approaches, the training set consists of I=8 observations (runs), and the available number of predictors *T* varies in each approach. In the first-order MLRM, we assume that the response and 4 predictors have a direct relation, such that there is no interaction or second-order term (T=4). The second-order MLRM includes the squared values of the predictors when fitting to the regression (T=8). The MLRM with interaction terms incorporates the pairwise interactions of the predictors (T=10) to the linear regression model. We considered MLRMs that have a maximum of 4 predictors (J=4), because the number of data was limited, and a better understanding of the impact of the predictors without forming excessively complex relations was desired. In fact, even without considering more than 4 predictors in each model, many models had predictors whose coefficients were statistically insignificant (*p*-value > 0.01) due to the limited data set, as is discussed below. Our analysis was carried out on R 4.0.3 using the lm function to fit the MLRMs [60]. We utilized the ggplot2 package to plot the selected MLRM [61] (see Section S2 in the Supplementary Materials for the R code).
**Algorithm 1:** Subset Selection1: Input: Training Data: ${\left(x_{i}^{1},\;x_{i}^{2},\;\ldots ,\;x_{i}^{T};y_{i}\right)}_{i=1}^{I}$
2: For each j=1, 2, …, J:
3: (a) Fit linear regression model for all combinations $\left(\begin{array}{c} T\\ j \end{array}\right)$ of predictors.4: (b) **Set the best** model *BM_j_* as the one with the highest coefficient of determination *R*^2^5: Select overall best models (BMs) among *BM*_1_,*BM*_2_,…,*BM_J_* as the one(s) that have adjusted *R*^2^ ≥ 0.99 and in which all predictors have a statistically significant relationship (*p value* ≤ 0.01) with the response

Aside from the kinetic–microhydrodynamic model, a purely empirical correlation between *k* and the process parameters/bead properties of the general form *k* = *k* (*ω*, *c*, *ρ*_b_, *Y*_b_) was sought using the subset selection algorithm. As this correlation does not use any microhydrodynamic variables that directly connect with some aspect of the bead–bead collisions, we refer to it as the purely empirical correlation.

## 3. Results and Discussion

### 3.1. Elucidation of the Particle Change Mechanisms

**Figure 1:**
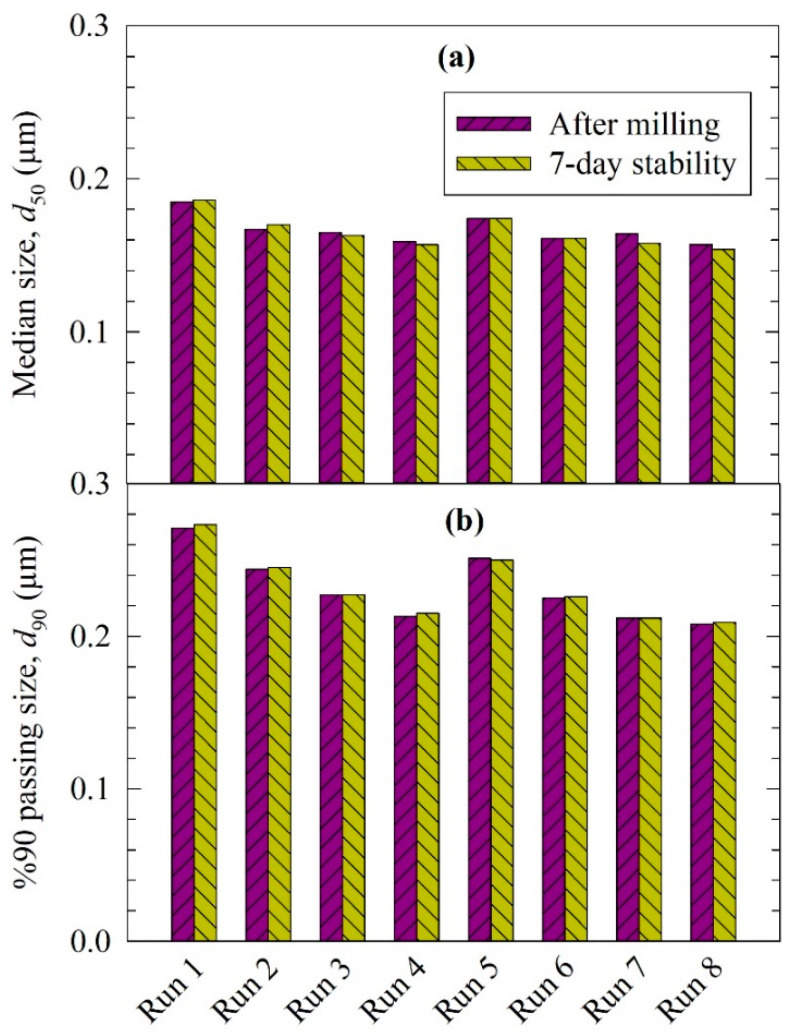
Volume-based particle size statistics of the milled FNB suspensions after milling (180 min) and after 7-day storage at 8 °C: (**a**) median particle size *d*_50_, and (**b**) 90% cumulative passing size *d*_90_.

Figure 1 presents the particle sizes in the FNB suspensions after 180 min milling and after 7-day refrigerated storage (Runs 1–8, DOE runs). The median sizes d_50_ were all below 200 nm, and the 90% volume passing sizes d_90_ were all below 300 nm. As the particle sizes did not change significantly after 7-day storage, all milled FNB suspensions were physically stable, and the impact of nanoparticle aggregation and/or particle growth through ripening processes was negligible. As will be shown in Section 3.2, the timewise evolutions of the d_50_–t profiles were all monotone-decreasing, which also confirms the mitigation of particle aggregation/growth to a large extent. Hence, the requirements (refer to Section 2.2.3) for an accurate analysis of the breakage kinetics were generally satisfied by our experiments; the particle breakage was the dominant particle change mechanism during the WSMM runs.

**Figure 2:**
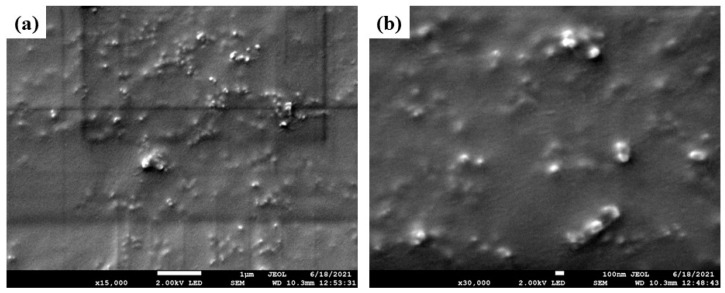
SEM image of 180 min milled FNB particles in Run 10 (*ω* = 3500 rpm, *c* = 0.425, and YSZ beads): (**a**) ×15 k magnification (scale bar: 1 µm), and (**b**) ×30 k magnification (scale bar: 100 nm).

Earlier work on the WSMM of FNB suspensions stabilized by hydroxypropyl methylcellulose (HPMC) and SDS revealed critical insights about the roles of the stabilizers [35,36]: excessive HPMC was required to suppress FNB nanoparticle aggregation in the absence of SDS; an optimal concentration of SDS exists (~0.05% *w*/*w*), which minimizes aggregation without facilitating Ostwald ripening. The ripening process was rather slow (occurred over days) and quite dependent on the SDS concentration, as the solubility of FNB increased with an increasing SDS concentration [38]. Knieke et al. [36] demonstrated, via laser diffraction and SEM imaging of the milled/stored fenofibrate nanoparticles in suspension form, that the ripening process had no impact on the milled particle size during the milling timescale at the SDS concentration range of 0.05–0.25% *w*/*w*. Only at 0.25% *w*/*w* SDS and after 7-day storage were notable particle growth (significant increase in d_50_ and d_90_) and the formation of new rhombohedron-shaped crystals reported [36]. Considering that our current study used 0.05% *w*/*w* SDS, based on Knieke et al. [36] alone, one would not expect that the ripening process would have any effect on the breakage kinetics. Indeed, the monotone-decreasing profile of d_50_ during the milling (Section 3.2), the invariance of d_50_ and d_90_ during the 7-day storage (Figure 1), and the absence of large rhombohedron-shaped drug crystals in the SEM images (Figure 2) clearly refute the notion that aggregation or Ostwald ripening played any significant role during the milling/storage.

Apparently, a 7.5% HPC/0.05% SDS combination mitigated nanoparticle aggregation without facilitating Ostwald ripening. Such neutral polymer/anionic surfactant combinations have been successfully used to stabilize a multitude of drug nanosuspensions, and their success has been attributed to an electrosteric stabilization mechanism as well as to the enhanced wettability of the relatively hydrophobic drug and the deaggregation of drug nanoparticle clusters during the milling [34,35,36,39,62,63]. Moreover, aside from their steric stabilizing action upon adsorption on drug nanoparticles, the cellulosic polymers such as HPC and HPMC are well known to inhibit nucleation and/or crystal growth (see e.g., [64]), which could have helped to mitigate the Ostwald ripening [36].

**Figure 3:**
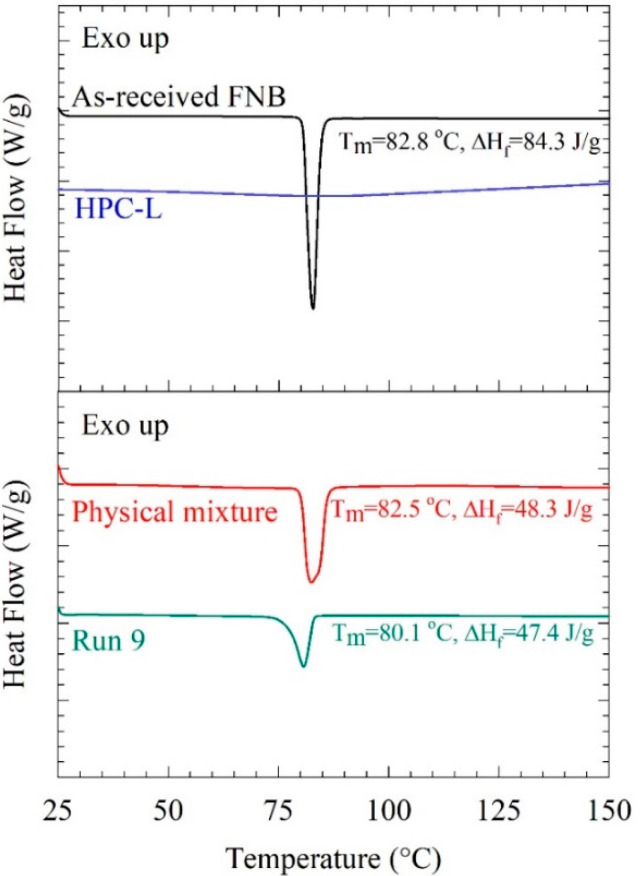
DSC traces, with the fusion enthalpy ΔH_f_ and the peak melting point temperature T_m_, of as-received FNB, HPC-L, the unmilled physical mixture, and the dried nanosuspension prepared in Run 10 (*ω* = 3500 rpm, *c* = 0.425, and YSZ beads).

Milling can change the solid state of a material due to mechanically induced defects and disorder of the crystal lattices, and even cause conversion of the crystalline material to an amorphous form [8,10,11]. The DSC traces in Figure 3 depict an endothermal event (fusion) for the as-received crystalline FNB particles, while such an event was absent from the amorphous polymer (HPC). The fusion enthalpy of FNB was reduced in approximate proportion to the amount of amorphous HPC in the physical mixture and the dried nanosuspension. The comparison of the DSC traces of the physical mixture and the dried nanosuspension reveals a 2.4 °C melting point depression, which could be attributed to the nanocrystalline nature of the FNB and the presence of mechanically induced defects.

### 3.2. Kinetic Analysis Via First-Order, nth-Order, and Warped-Time Models

**Figure 4:**
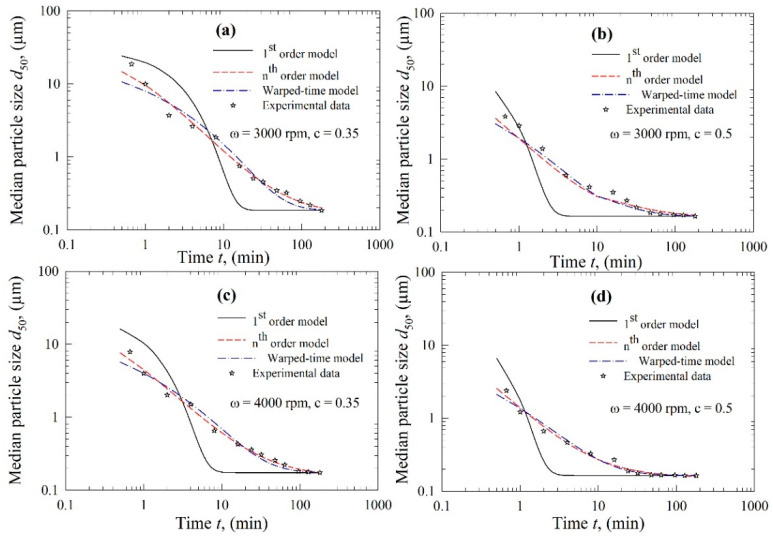
Temporal evolution of the median particle size d_50_ during the wet milling of fenofibrate with CPS beads and fitting of the data by various kinetic models: (**a**) *ω* = 3000 rpm and *c* = 0.35, (**b**) *ω* = 3000 rpm and *c* = 0.5, (**c**) *ω* = 4000 rpm and *c* = 0.35, and (**d**) *ω* = 4000 rpm and *c* = 0.5.

**Figure 5:**
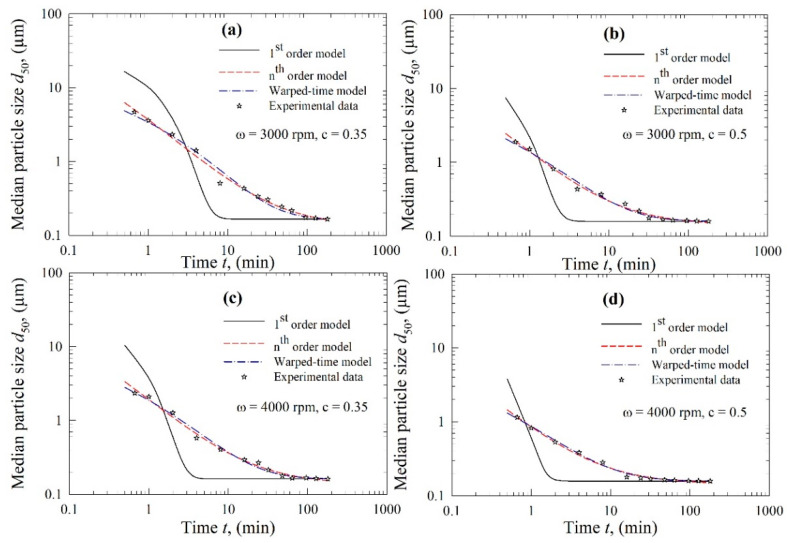
Temporal evolution of the median particle size d_50_ during the wet milling of fenofibrate with YSZ beads and fitting of the data by various kinetic models: (**a**) *ω* = 3000 rpm and *c* = 0.35, (**b**) *ω* = 3000 rpm and *c* = 0.5, (**c**) *ω* = 4000 rpm and *c* = 0.35, and (**d**) *ω* = 4000 rpm and *c* = 0.5.

The kinetics of FNB particle breakage during WSMM were examined using three kinetic models, i.e., the first-order model, the *n*th-order model, and the warped-time model. To discriminate these models and identify the best model, the experimental temporal evolution of the median size *d*_50_ and the fittings of the models are presented for CPS beads and YSZ beads in Figure 4 and Figure 5, respectively. Figure 4 and Figure 5 show that the coarse FNB particles break much faster (within the first 2–10 min) than the particles in the colloidal size range (<1 µm). It should be noted that the time axis is logarithmic; the median size decreased monotonically over time, and tended toward or attained a limiting size, which is the typical dynamic behavior for a well-stabilized suspension, confirming the judicious selection of the stabilizers based on our earlier studies. As the impact of particle aggregation and growth was negligible (refer to Section 3.1), these observations can be explained by slowing breakage kinetics during WSMM, which can be attributed to the higher strength of the finer particles than the coarser particles, and the reduced probability of capturing the finer particles between the beads [27,30,34].

Regardless of the used bead material, the trends in Figure 4 and Figure 5 also suggest that the first-order kinetics model failed to fit the experimental data for all conditions. This observation was supported by the fitting statistics in Table 2, where the model parameters were found to be statistically significant (*p*-value ≤ 0.01); however, the fitting was poor, with adjusted R^2^ < 0.90 for all conditions. Even though the first-order kinetics model is popular, as discussed previously, it was inadequate to represent the entirety of the experimental kinetic data governed by two or potentially more breakage rate constants [44]. As we wanted to perform a fair, head-to-head comparison of all of the models, all experimental data collected were used in the parameter estimation. While the fitting may be improved by removing some of the initial data points [33,34], this would come at the expense of reduced robustness of the model. Therefore, no experimental data were disregarded here.

The first-order model performed poorly compared to the *n*th-order and the warped-time models (refer to Figure 4 and Figure 5). The latter two models followed the experimental breakage trends very well. The fitting statistics presented in Table 3 and Table 4 confirm that the *n*th-order model and the warped-time model had both statistically significant parameters, and their fitting capability was excellent: adjusted R^2^ ≥ 0.99, except for the fitting of the Run 1 data by the warped-time model. When all 12 runs were considered, the *n*th-order model fitted the experimental data better than the warped-time model, as can be inferred from the higher R^2^ and adjusted R^2^, as well as the lower sum-of-squared residuals (SSR) (except for Runs 8 and 12).

### 3.3. Effects of Process Variables on the Kinetic Parameters

**Figure 6:**
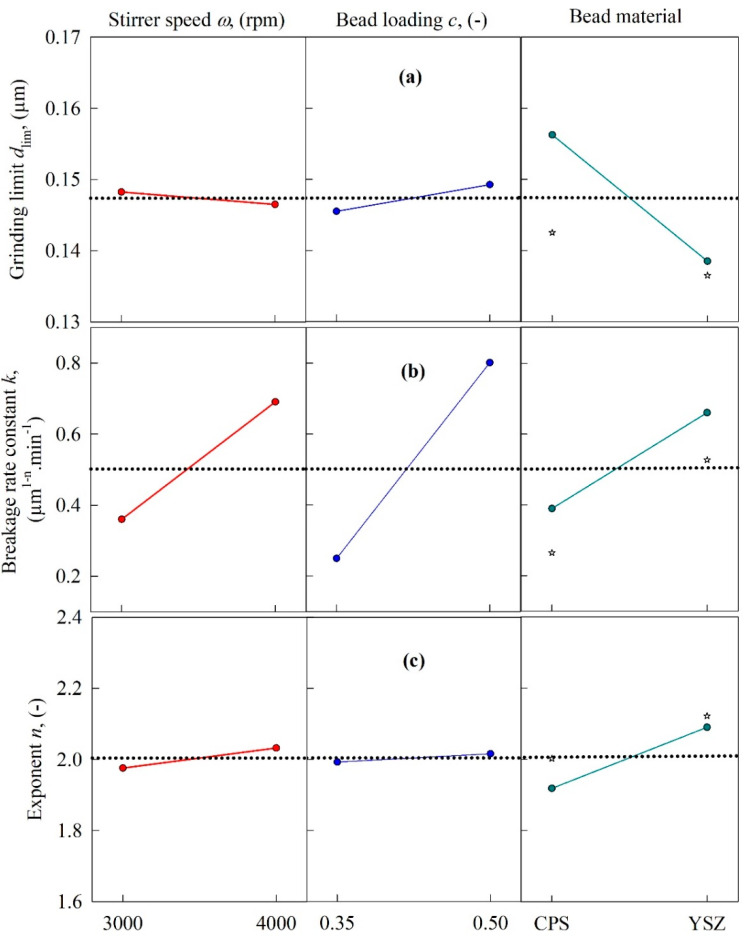
Main effects plots for the parameters of the *n*th-order kinetics model as a function of the following process variables: (**a**) grinding limit *d*_lim_, (**b**) breakage rate constant *k*, and (**c**) exponent *n.* The center point experimental data (Runs 9 and 10) were added to the rightmost panel.

As both the *n*th-order model and the warped-time model were found to have excellent fitting capability, the impact of process variables on their model parameters—i.e., the limiting size *d*_lim_, the breakage rate constant *k* or *k*_0_, and the exponent *n*—was explored. For the *n*th-order model parameters, the main effects plots in Figure 6 and the fitted parameters in Table 3 (DOE Runs 1–8) suggest the following major trends: (1) the breakage rate constant *k* was significantly higher at higher stirrer speeds, higher bead loading, and with the use of the YSZ beads as opposed to the CPS beads; (2) *d*_lim_ varied in a narrow range from 132 to 161 nm, with an 8-run average of 148 ± 11 nm (RSD = 7.4%); it decreased slightly at higher speeds and with the use of the YSZ beads, and increased slightly at higher bead loading; (3) *n* slightly increased upon an increase in the stirrer speed and the use of the YSZ beads, and the bead loading had almost no effect. The changes were so small that the breakage kinetics were nearly second-order overall (*n* = 2.00 ± 0.06 from an 8-run average, RSD = 3.0%). As compared to the drastic variation in *k*, the variation in *n* and *d*_lim_ for a given bead material (CPS or YSZ) was relatively small, thus justifying the use of constant, average values of *n* and *d*_lim_ for a specific bead material.

**Figure 7:**
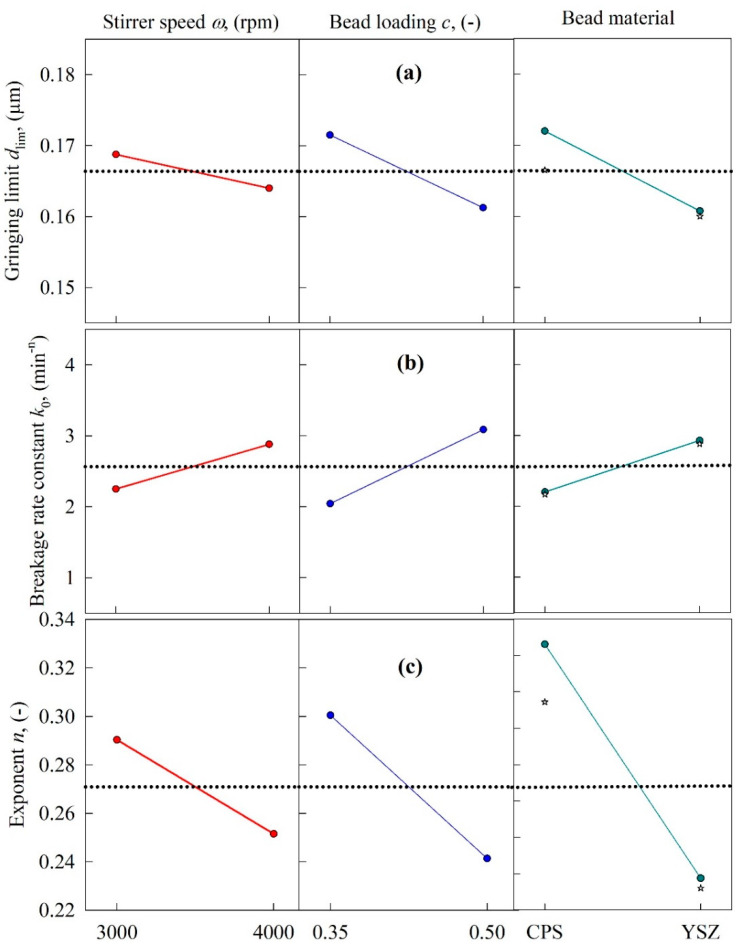
Main effects plots for the parameters of the warped-time kinetics model as a function of the following process variables: (**a**) grinding limit *d*_lim_, (**b**) breakage rate constant *k*_0_, and (**c**) exponent *n*. The center point experimental data (Runs 9 and 10) were added to the rightmost panel.

The variation of the breakage rate constant *k*_0_ of the warped-time model with the process parameters (see Figure 7 and refer to Table 4) was similar to that of *k* of the *n*th-order model. However, both *d*_lim_ and *n* were lower at higher stirrer speeds, higher bead loading, and with the use of the YSZ beads as opposed to the CPS beads. While *d*_lim_ again varied in a narrow range from 157 to 185 nm with an 8-run average of 166 ± 9 nm, and the impact of the process parameters was rather limited (RSD = 5.4%), the relative change in *n* was notable for different processing conditions: 8-run average of 0.271 ± 0.03 (RSD = 11%).

Although the warped-time model has excellent fitting capability, two issues warrant discussion: First, the grinding limit *d*_lim_ was found to be equal to the final milled particle size at 180 min, which is somewhat unrealistic, as in the limit *t*→∞ the limiting particle size must be smaller than that at 180 min. This was correctly captured by the smaller *d*_lim_ of the *n*th-order model (see Table 3 vs. Table 4). Second, unlike the case for *k* and *n* of the *n*th-order model, both k_0_ and n drastically changed in opposite directions, and considering the time-dependence of$\;k\left(t\right)=k_{0}nt^{n-1}$, the impact of the processing variables on the overall breakage rate is hard to interpret without further quantitative analysis. Along with the better fitting capability of the *n*th-order model, these physical considerations led us to choose the *n*th-order model as the best kinetic model, and to use it for the microhydrodynamic correlations. If we were to choose the warped-time model, there would be two separate kinetic–microhydrodynamic correlations—one for *k* and another for *n*—and this would clearly be an undesirable situation.

Figure 6 and Figure 7 (rightmost panel) show that the center point responses (Run 9 for CPS and Run 10 for YSZ) and the mean values for CPS (Runs 1, 3, 5, and 7) and YSZ (Runs 2, 4, 6, and 8) deviated, although they yielded similar trends in terms of increase/decrease of the kinetic parameters upon use of the CPS beads vs. the YSZ beads. We have not used the main effects plots to establish a quantitative model between the kinetic parameters and the process parameters; the kinetic–microhydrodynamic correlation of the form *k* = *k* (*a*, *σ*_b_^max^, *α*_b_, *Πσ*_y_) implicitly achieves that task (see Section 3.4). As will also be shown in Section 3.4, the purely empirical correlation of the form *k* = *k* (*ω*, *c*, *ρ*_b_, *Y*_b_) was not linear; there exist several interactions among the independent variables.

### 3.4. Microhydrodynamic Origin of the Calculated Breakage Rate Constant

**Figure 8:**
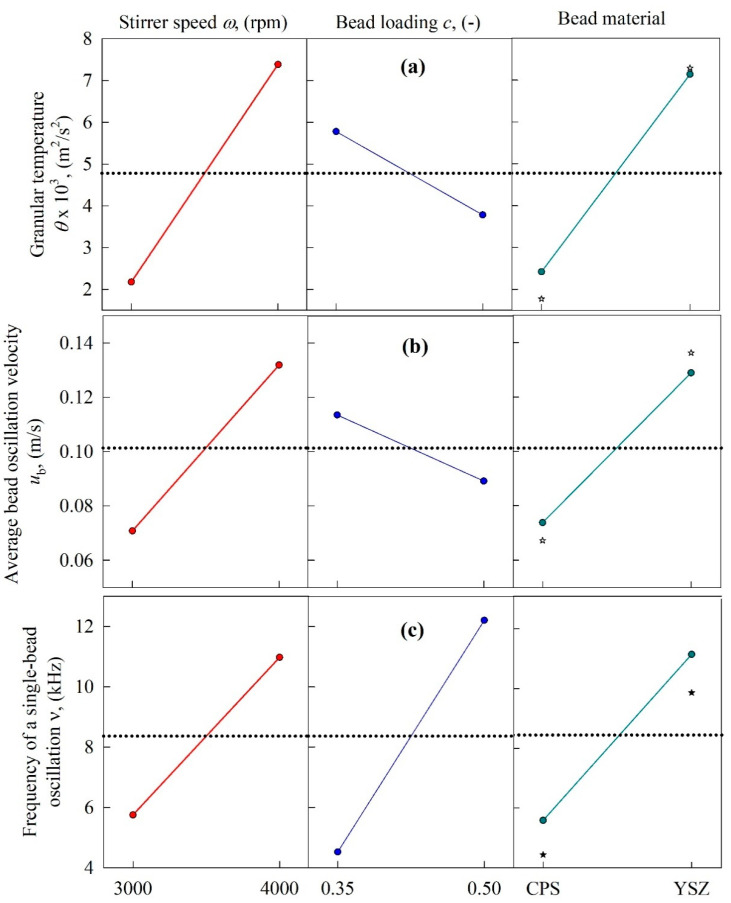
Main effects plots for the microhydrodynamic parameters as a function of the following process variables: (**a**) granular temperature *θ*, (**b**) average bead oscillation velocity *u*_b_, and (**c**) frequency of a single-bead oscillation *ν*. The center point experimental data (Runs 9 and 10) were added to the rightmost panel.

**Figure 9:**
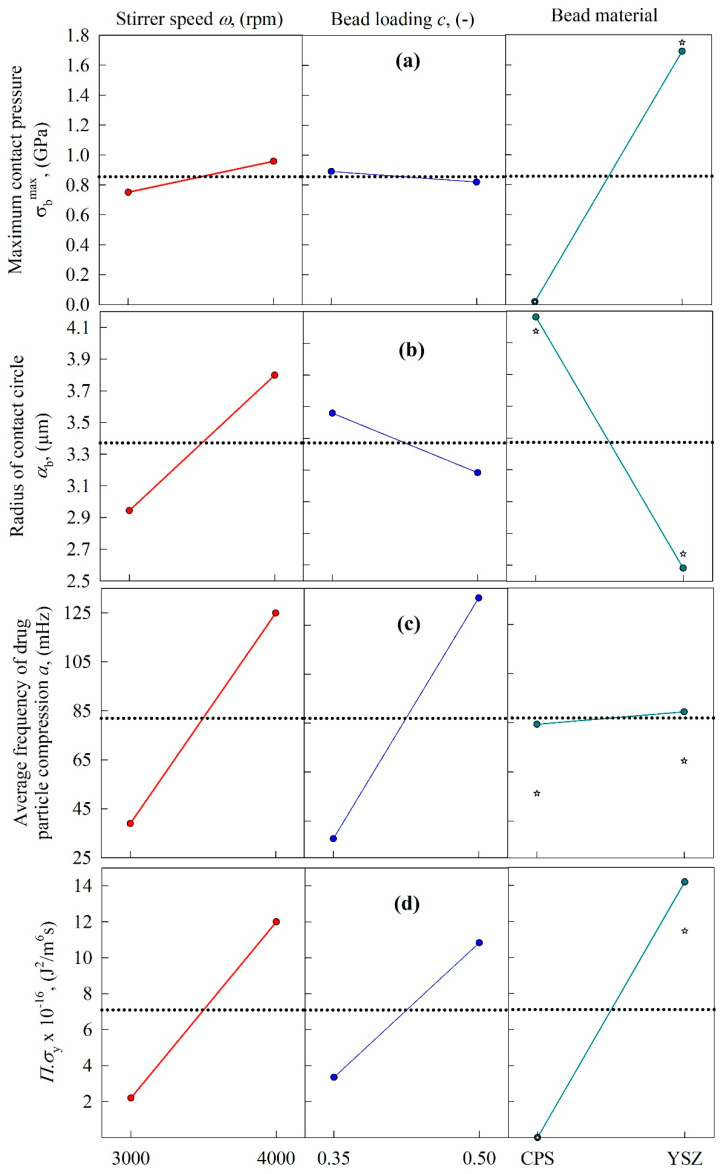
Main effects plots for the microhydrodynamic parameters as a function of the following process variables: (**a**) maximum contact pressure *σ*_b_^max^, (**b**) radius of contact circle *α*_b_, (**c**) average frequency of drug particle compression *a*, and (**d**) the pseudo energy dissipation rate *Πσ*_y_. The center point experimental data (Runs 9 and 10) were added to the rightmost panel.

The microhydrodynamic parameters—i.e., granular temperature *θ,* average bead oscillation velocity *u*_b_, frequency of a single-bead oscillation *ν*, maximum contact pressure *σ*_b_^max^, radius of contact circle *α*_b_, average frequency of drug particle compressions *a*, and pseudo energy dissipation rate for the drug particles *Πσ*_y_—for all 8 runs were calculated and presented in Table S1 of the Supplementary Materials. The main effects plots (Figure 8 and Figure 9) and Table S1 show that all of the microhydrodynamic parameters were significantly higher at higher stirrer speeds, signifying more frequent collisions of the beads with higher stress intensity, and ensuring higher frequency of drug particle compressions. This is the microhydrodynamic origin of the higher breakage rate *k* at higher stirrer speeds (refer to Figure 6 and Table 3). The use of the YSZ beads as opposed to the CPS beads also led to significantly higher microhydrodynamic parameters, due to the much higher density of the YSZ beads—albeit with two notable exceptions (Figure 8 and Figure 9). Owing to their lower modulus of elasticity, CPS beads had higher *α*_b_ than the YSZ beads (Figure 9b); hence, the CPS beads could capture more drug particles per CPS–CPS bead collision. This effect was counteracted by the higher *θ*, *u*_b_, *ν*, *σ*_b_^max^, and *Πσ*_y_ of the YSZ beads, signifying a higher number of more energetic/forceful YSZ bead–bead collisions. These two counteracting effects led to a slight increase in the frequency of drug particle compressions *a* when the YSZ beads vs. the CPS beads were used (Figure 9c), which favored breakage (refer to Figure 6 and Table 3). We also note from Figure 8 and Figure 9 (rightmost panel) that the center point responses (Run 9 for CPS and Run 10 for YSZ) and the mean values for CPS (Runs 1, 3, 5, and 7) and YSZ (Runs 2, 4, 6, and 8) yielded similar trends in terms of increase/decrease of the microhydrodynamic parameters upon use of CPS vs. YSZ.

When a higher bead loading *c* was used, two counteracting effects were observed (Figure 8 and Figure 9). Due to the occurrence of higher drag forces and more bead–bead squeezing events at the higher *c*, the energy dissipation due to viscous losses and inelastic collisions was higher, which led to lower *θ*, *u*_b_, *α*_b_, and *σ*_b_^max^ (lower energy/less forceful bead collisions), and did not favor particle breakage. However, the dramatic increase in the concentration of the beads, along with higher *g*_0_, led to more frequent collisions, signified by higher *ν*, *a*, and *Πσ*_y_, which favors breakage. It appears that higher *ν*, *a*, and *Πσ*_y_ (favorable for breakage) are much more influential on *k* than lower *θ*, *u*_b_, *α*_b_, and *σ*_b_^max^ (unfavorable for breakage), as inferred from the positive impact of *c* on *k* (refer to Table 3 and Figure 6).

A statistically and physically significant relationship between the microhydrodynamic parameters and the breakage rate constant *k* was expected based on the microhydrodynamic insights, and was examined via multiple linear regression models (MLRMs). Three different MLRM approaches were investigated, i.e., the first-order MLRM, where the microhydrodynamic parameters are considered to have a linear relationship with *k*; the second-order MLRM, where the squares of the microhydrodynamic parameters are also considered to have an impact on *k*; and the MLRM with interaction terms, where the multiple of two microhydrodynamic parameters may affect *k*. On purely physical grounds, *k* must be zero if any microhydrodynamic parameter is zero, suggesting a zero intercept. Indeed, when the intercept was included in the models, one or more MLRM coefficients—including the intercept—were generally found to be statistically insignificant (see Table S2 in the Supplementary Materials). Hence, the intercept was set to zero in the models. We selected *σ*_b_^max^, *α*_b_, *a*, and *Πσ*_y_ as the predictive microhydrodynamic parameters, since they are not directly correlated with one another, and each of them represents a different aspect of the bead–bead collisions (*σ*_b_^max^ and *α*_b_) and the compression frequency/energy of the captured drug particles (*a* and *Πσ*_y_).

As the maximum allowed number of predictors *J* was chosen as four, the algorithm considers the four best models (*BM*_1_, *BM*_2_, *BM*_3_, and *BM*_4_) for each MLRM. As can be seen from Table 5, *BM*_1_ has the average frequency of drug particle compression *a* as the predictor, with an adjusted R^2^ of 0.91 for all three MLRMs. *BM*_2_ was found to have the same two predictors for all MLRMs, where maximum contact pressure *σ*_b_^max^ and *a* were again found to have the most impact on *k*. For *BM*_3_, the algorithm selected the radius of the contact circle *α*_b_ in addition to the predictors used in *BM*_2_ when the first-order MLRM was used. On the other hand, when the second-order MLRM was used, the algorithm selected *a*^2^ in addition to the predictors used in *BM*_2_ for *BM*_3_. Moreover, *BM*_3_ using the MLRM model with interaction terms suggests that *a* should be used together with its interaction with *α*_b_ and *Πσ*_y_. For *BM*_4_, the selected predictors contain all available predictors for the first-order MLRM. For the second-order MLRM, the algorithm removed *σ*_b_^max^ from *BM*_3_ and added *Πσ*_y_ and *α*_b_^2^ for *BM*_4_. Finally, for the MLRM with interaction terms, the algorithm kept all *BM*_3_ predictors for *BM*_4_, with the addition of *Πσ*_y_. To select the best model among all of the models presented in Table 5, the following criteria were used: adjusted R^2^ ≥ 0.99 and *p*-value ≤ 0.01. Only the following three-parameter MLRM with interaction terms satisfied the criteria:

(17) $$k=1.87\times 10^{-2}a-3.25\times 10^{-3}\alpha_{b}a-9.77\times 10^{-5}\left(\Pi \sigma_{y}\right)a$$

**Figure 10:**
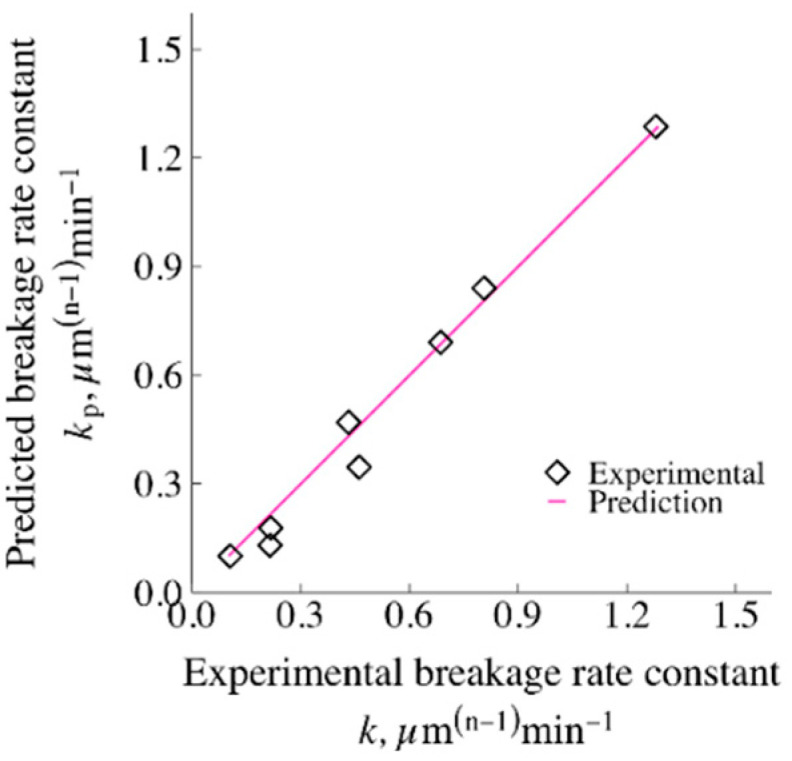
The breakage rate parameter *k* of the *n*th-order model predicted using the 3-parameter MLRM with interaction terms (Equation (17)), vs. the experimentally determined *k*. This is the only MLRM that satisfied adjusted R^2^ ≥ 0.99 and *p*-value ≤ 0.01 for all coefficients.

The *k* predicted by Equation (17) vs. the actual *k* of the *n*th-order kinetics model is presented in Figure 10. Overall, these results corroborate that *a* is the most important microhydrodynamic parameter, which explains most of the process-related variation of the breakage rate constant *k*, along with its interaction with *α*_b_ and *Πσ*_y_. 

To gauge the usefulness of the proposed kinetic–microhydrodynamic correlation in Equation (17), we compared it to a purely empirical correlation, which does not require any fundamental understanding of the underlying microhydrodynamics. The breakage rate constant was simply assumed to be a function of the process parameters *ω* and *c,* as well as the bead properties *ρ*_b_ and *Y*_b_, i.e., *k* = *k*(*ω*, *c*, *ρ*_b_, *Y*_b_). The bead properties were different for different bead materials. The statistical results from the MLRM and the subset selection algorithm are presented in Table 6.

As most MLRMs for the purely empirical correlation have relatively low R^2^, high SSR, and mostly statistically insignificant parameters (at the 99% confidence level), we relaxed the statistical significance criterion from the 99% confidence level to the 95% confidence level—i.e., *p* ≤ 0.05, which has also been used widely in the literature. Hence, *BM_4_* with the interaction terms was selected, as it satisfied adjusted R^2^ ≥ 0.99 with all statistically significant coefficients at the 95% confidence level. This purely empirical correlation—Equation (18)—signifies multiple binary interactions among the independent variables:

(18) $$k=-3.01\times 10^{-4}\omega +1.50\times 10^{-3}\omega c-1.87\times 10^{-3}c\rho_{b}+5.02\times 10^{-2}cY_{b}$$

### 3.5. Predictive Capability of the Kinetic–Microhydrodynamic Model and the Purely Empirical Model

**Figure 11:**
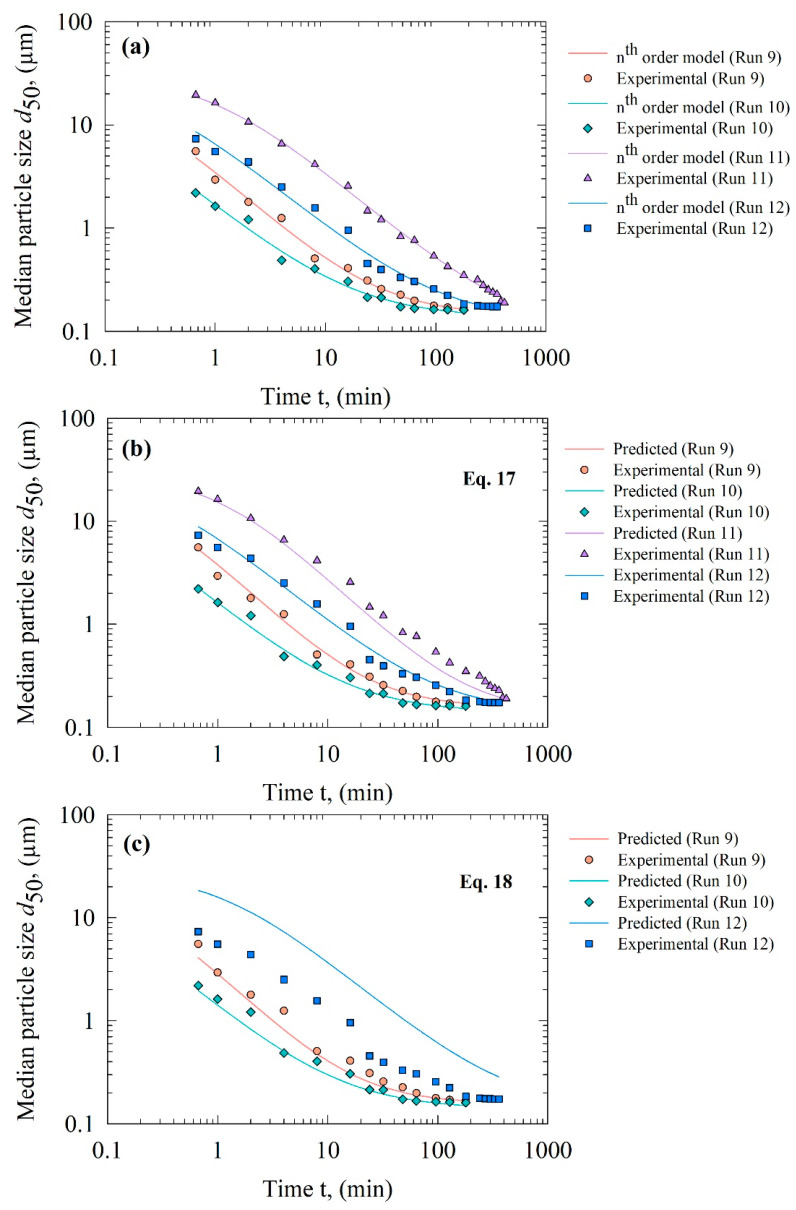
Temporal evolution of the median particle size *d*_50_ during the wet milling of fenofibrate, and (**a**) its direct fit by the *n*th-order kinetics model in Equation (3), (**b**) the predicted evolution of *d*_50_ using *k* estimated by Equation (17), and (**c**) the predicted evolution of *d*_50_ using *k* estimated by Equation (18).

The timewise evolution of the median size in Runs 9–12—which were not used in the calibration of the kinetic–microhydrodynamic correlation and the purely empirical correlation—was first directly fitted using the *n*th-order kinetics model (Figure 11a). Table 7 presents the statistical summary; as expected, Figure 11a and Table 7 show that the kinetic model fitted the data well (R^2^ > 0.99). Then, we estimated the *k* value of the *n*th-order model using the kinetic–microhydrodynamic correlation (Equation (17)) and the purely empirical correlation (Equation (18)). As the *n* and *d*_lim_ values varied in a much smaller range than the *k* values for the DOE (Runs 1–8), in the predictions, we assumed constant values for *n* and *d*_lim_ by calculating their average values for the CPS beads (Runs 1, 3, 5, and 7) and the YSZ beads (Runs 2, 4, 6, and 8). A comparison of these average *n* and *d*_lim_ values in the predictions vs. the directly fitted *n* and *d*_lim_ values for Runs 9–12 (Table 7) reveals that this assumption was generally valid, and the deviations from the fitted values were reasonably small, with the sole exception of Run 11’s *d*_lim_. The relatively low estimated *d*_lim_ value was most likely related to the fact that even after 7 h of milling, the profile did not attain or approach a plateau under the lowest energetic conditions with the CPS beads. In general, the alternative approach of developing correlations for *n* and *d*_lim_ similar to those for *k* appears to be unwarranted.

Figure 11b presents the temporal evolution of the median size in Runs 9–12 predicted by the kinetic–microhydrodynamic correlation (Equation (17)), while Figure 11c presents the same predictions by the purely empirical model (Equation (18)). A cursory look at Table 7 and visual assessment of the figures therein reveals that the purely empirical correlation did not even predict the evolution at the center point conditions (Runs 9 and 10). The reason for this is that the empirical correlation requires many more data points or experimental milling runs: e.g., 18 runs for 3 levels (low–medium–high) of values of the stirrer speed and the bead loading with the CPS and YSZ beads, which would increase the resources, time, and effort by 125% compared to the current 8-run DOE. Such an expanded DOE could enable the empirical model to have statistically more reliable parameters, and perhaps additional terms in Equation (18) for better fitting capability. However, it is unclear whether that could resolve the second major issue with the purely empirical model, i.e., its utter failure to predict the profiles of Runs 10 and 11 with the experimental conditions outside the domain of the 8-run DOE; in fact, it predicts a negative *k*-value for Run 11 (no prediction curve presented in Figure 11c). Hence, as expected, the purely empirical model had little to no predictive capability. In contrast, the kinetic–microhydrodynamic correlation has remarkable prediction capability for Runs 9 and 10, as signified by its R^2^ > 0.99 and low SSR values that are close to those of the direct fitting. Similarly excellent prediction was made for Run 12, which was outside the DOE. Although the kinetic–microhydrodynamic model underpredicted the median size after 10 min of milling in Run 11, this prediction was reasonable, and certainly superior to that by the purely empirical model, which predicted a negative *k*.

### 3.6. Limitations of the Models

The main assumptions and limitations of the microhydrodynamic model have been mentioned in Section 2.2.4; here, we focus on those related to the kinetic models. In the DOE, we considered a fixed batch size and suspension flow rate, and investigated the impact of the stirrer speed, bead loading, and bead type. Hence, the models in this study are strictly valid only at the respective experimental scale/batch size for the given flow rate in the recirculation mill. In the recirculation mill, two separate characteristic times exist: the mean residence time in the mill (*τ*_m_ = *V*_sm_/*Q*), where *Q* is the suspension flow rate and *V*_sm_ is the suspension volume in the mill; and the mean residence time in the holding tank (*τ*_T_ = *V*_T_/*Q*), where *V*_T_ is the volume (or batch size) in the holding tank. Hence, the milled particle-size distribution (PSD) and the overall breakage kinetics depend on these two characteristic times. Unfortunately, our simple kinetics models cannot rigorously capture the impact of recirculation. A rigorous analysis of the recirculation could be made using a population balance model (PBM) for both the mill and the holding tank, by assuming well-mixedness or determining the residence time distribution experimentally. Interestingly, even most of the existing PBMs for the recirculation mode of WSMM used equations valid for a batch mill, without any consideration of the holding tank or the two different mean residence times (e.g., [65,66]). We found two PBM studies that correctly accounted for the recirculation effects [67,68]; however, Diemer [68] did not consider WSMM, while Annapragada and Adjei [67] used a specific breakage-rate kernel that incorporated an unrealistic physical model (see [26]). To the best knowledge of the authors [26], a PBM that incorporates underlying microhydrodynamic parameters does not exist.

In this study, we did not investigate the impact of the batch size or the suspension flow rate, which can be rigorously examined using a PBM. The batch size is typically set—it has rarely been varied and examined in the pharmaceutical WSMM literature [22]. A comparison of the first-order time constants estimated for the breakage kinetics of identical griseofulvin suspensions under nearly identical process conditions in the same recirculation mill [9,34] suggests that the particles were coarser at any given time when the batch size was 440 mL vs. 220 mL, except when the milling time was long enough for the particles to reach the limiting size. At any milling time, the number of theoretical passes of the holding tank content through the mill was halved (mean residence time doubled) when the batch size was doubled. For a fixed batch size, an increase in the suspension flow rate led to a higher number of theoretical passes of the entire mill content through the mill, as well as to a lower mean residence time both in the mill and in the holding tank, which was found to result in finer particles [67] and a sharper PSD at a given time [9,67].

### 3.7. Future Perspectives and Outlook for Various Uses of the Kinetic–Microhydrodynamic Model

In this study, excellent descriptive (fitting) capability of the *n*th-order kinetics model, as well as the good predictive capability of the kinetic–microhydrodynamic correlation, has been established. Not only do these models provide mechanistic process understanding of the bead–bead collisions and capture frequency of the drug particles in the mill, but they also enable engineers to predict the evolution of the median particle size for a multitude of stirrer speed/bead loading/bead type combinations. This could save effort, time, and materials, as well as allow engineers to identify failure modes and optimal processing conditions and bead types. However, the current kinetic–microhydrodynamic correlation is not independently predictive, as the power consumption data needed for Runs 9–12 were still obtained from the experiments. To obviate the need for measuring the power consumption *P*_w_ without too many additional experiments, one can use the *P*_w_ values obtained from the DOE (Runs 1–8) and develop a general correlation for *P*_w_ as a function of the dimensionless Euler (power) number Eu, Reynolds number Re, and Froude number Fr for a specific mill, and such a correlation can be used for predicting *P*_w_ [69]. Alternatively, *P*_w_ can be estimated by a relatively inexpensive Eulerian–Eulerian simulation—with kinetic theory of granular flow (KTGF) for the beads phase (see e.g., [70])—of the torque required to rotate the stirrer of the mill via computational fluid dynamics (CFD), and this approach can be validated using the existing data from Runs 1–8. Such simulations would also allow for a thorough understanding of the recirculation effects. As discussed in Section 3.7, the impact of recirculation on the particle size can be analyzed using a PBM for the mill/holding tank. Clearly, there is a strong need for a mechanistic PBM that incorporates the microhydrodynamic parameters in its specific breakage rate, which is analogous to *k* here. Hence, the current study and the establishment of the *k* correlation with the three microhydrodynamic parameters motivate the development of a mechanistic PBM.

## 4. Conclusions

This study has examined the FNB breakage rate in WSMM operating at various stirrer speeds and volumetric loadings of CPS–YSZ beads via three kinetic models. The newly developed *n*th-order kinetics model with *n* ≅ 2 turned out to be the best overall model, which described the temporal evolution of the median size well. While the emergence of nearly second-order kinetics is interesting, further research is warranted to ascertain whether this finding has general applicability to multiple drugs and a broader range of processing conditions. The breakage rate constant *k* of this model was found to be higher at higher stirrer speeds, bead loading, and with use of the YSZ vs. CPS beads. Hence, cycle time will be greatly reduced by running the WSMM at 4000 rpm with 50% YSZ beads. The microhydrodynamic parameters provided valuable insights and a physical basis for the observed breakage behaviors under different operating conditions. Using a subset selection algorithm, a statistically significant (*p* ≤ 0.01) kinetic–microhydrodynamic correlation for *k* as a function of three microhydrodynamic parameters was found, which best explained the variation in the breakage rate constant (R^2^ ≥ 0.99). The average frequency of drug particle compressions interacting with two other microhydrodynamic parameters—i.e., pseudo energy dissipation rate and the radius of the bead–bead contact circle—govern the breakage kinetics in WSMM. We have also developed a purely empirical correlation for *k* as a function of the stirrer speed/bead loading/bead properties. The kinetic–microhydrodynamic model had good predictive capability overall, whereas the purely empirical model failed in its predictions. These kinetic models and the kinetic–microhydrodynamic correlation are expected to be useful to pharmaceutical engineers, who can (1) describe the breakage kinetics for the WSMM process quantitatively; (2) gain advanced process understanding and insights into the development of a WSMM process with reduced cycle time; and (3) predict the evolution of the median particle size for a multitude of stirrer speed/bead loading/bead type combinations, thus saving effort, time, and materials; and (4) potentially identify failure modes and optimal processing conditions/bead types. This study also hints at the need for performing CFD simulations to predict the power consumption, and for developing a microhydrodynamically inspired population balance model (PBM) to predict the timewise evolution of the whole-drug particle-size distribution (PSD)—not just the median particle size—and elucidate the impact of recirculation during the WSMM.

## Figures and Tables

**Table 1:** Process variables and bead materials used in the wet media milling of the fenofibrate suspensions.

Run No.	Stirrer Speed, *ω* (rpm)	Bead Loading, *c* (-)	Bead Material
1 ^a^	3000	0.35	CPS
2 ^a^	3000	0.35	YSZ
3 ^a^	3000	0.50	CPS
4 ^a^	3000	0.50	YSZ
5 ^a^	4000	0.35	CPS
6 ^a^	4000	0.35	YSZ
7 ^a^	4000	0.50	CPS
8 ^a^	4000	0.50	YSZ
9 ^b^	3500	0.425	CPS
10 ^b^	3500	0.425	YSZ
11 ^c^	2550	0.298	CPS
12 ^c^	2550	0.298	YSZ

^a^: Three-factor, two-level, full factorial DOE used in the parameter estimation of the models. ^b^: Center points of the original DOE for the CPS and the YSZ beads. ^c^: Experiments whose variables are outside the domain of the DOE.

**Table 2:** Statistical summary of parameter estimation using the first-order kinetics model.

Run	Parameter	Value	*p*-Value	R^2^	Adj. R^2^	SSR
1	*d*_lim_ (µm)	0.185	0.0036	0.818	0.803	1.32
	*k* (min^−1^)	0.420	0.0006			
2	*d*_lim_ (µm)	0.166	0.0013	0.805	0.789	1.16
	*k* (min^−1^)	0.992	0.0002			
3	*d*_lim_ (µm)	0.165	<0.0001	0.866	0.854	0.786
	*k* (min^−1^)	1.70	<0.0001			
4	*d*_lim_ (µm)	0.159	0.0001	0.851	0.838	0.767
	*k* (min^−1^)	2.54	<0.0001			
5	*d*_lim_ (µm)	0.174	0.0009	0.832	0.818	1.04
	*k* (min^−1^)	0.91	0.0002			
6	*d*_lim_ (µm)	0.161	0.0005	0.820	0.805	0.994
	*k* (min^−1^)	2.09	<0.0001			
7	*d*_lim_ (µm)	0.164	<0.0001	0.884	0.875	0.600
	*k* (min^−1^)	2.80	<0.0001			
8	*d*_lim_ (µm)	0.157	<0.0001	0.889	0.880	0.554
	*k* (min^−1^)	4.17	<0.0001			
9	*d*_lim_ (µm)	0.166	0.0010	0.817	0.802	1.09
	*k* (min^−1^)	1.10	0.0002			
10	*d*_lim_ (µm)	0.161	0.0004	0.823	0.809	0.934
	*k* (min^−1^)	2.18	<0.0001			
11	*d*_lim_ (µm)	0.190	0.0001	0.838	0.830	1.728
	*k* (min^−1^)	0.115	<0.0001			
12	*d*_lim_ (µm)	0.174	0.0001	0.796	0.794	1.710
	*k* (min^−1^)	0.445	<0.0001			

**Table 3:** Statistical summary of parameter estimation using the *n*th-order kinetics model.

Run	Parameter	Value	*p*-Value	R^2^	Adj. R^2^	SSR
	*d*_lim_ (µm)	0.161	0.0015	0.989		0.077
1	*k* (µm^(*n* − 1)^min^−1^)	0.105	0.0002		0.987	
	*n* (-)	1.86	<0.0001			
	*d*_lim_ (µm)	0.132	<0.0001	0.995		0.029
2	*k* (µm^(*n* − 1)^min^−1^)	0.217	<0.0001		0.994	
	*n* (-)	2.06	<0.0001			
	*d*_lim_ (µm)	0.158	<0.0001	0.996		
3	*k* (µm^(*n* − 1)^min^−1^)	0.432	<0.0001		0.996	0.022
	*n* (-)	1.90	<0.0001			
	*d*_lim_ (µm)	0.142	<0.0001	0.997		0.013
4	*k* (µm^(*n* − 1)^min^−1^)	0.686	<0.0001		0.997	
	*n* (-)	2.08	<0.0001			
	*d*_lim_ (µm)	0.152	<0.0001	0.995		0.030
5	*k* (µm^(*n* − 1)^min^−1^)	0.215	<0.0001		0.994	
	*n* (-)	1.95	<0.0001			
	*d*_lim_ (µm)	0.137	<0.0001	0.997		0.016
6	*k* (µm^(*n* − 1)^min^−1^)	0.461	<0.0001		0.997	
	*n* (-)	2.10	<0.0001			
	*d*_lim_ (µm)	0.154	<0.0001	0.996		0.023
7	*k* (µm^(*n* − 1)^min^−1^)	0.806	<0.0001		0.995	
	*n* (-)	1.96	<0.0001			
	*d*_lim_ (µm)	0.143	<0.0001	0.998		0.009
8	*k* (µm^(*n* − 1)^min^−1^)	1.28	<0.0001		0.998	
	*n* (-)	2.12	<0.0001			
	*d*_lim_ (µm)	0.142	<0.0001	0.996		0.023
9	*k* (µm^(*n* − 1)^min^−1^)	0.264	<0.0001		0.995	
	*n* (-)	2.00	<0.0001			
	*d*_lim_ (µm)	0.136	<0.0001	0.996		0.023
10	*k* (µm^(*n* − 1)^min^−1^)	0.527	<0.0001		0.995	
	*n* (-)	2.12	<0.0001			
	*d*_lim_ (µm)	0.093	<0.0001	0.999		0.015
11	*k* (µm^(*n* − 1)^min^−1^)	0.023	<0.0001		0.998	
	*n* (-)	2.09	<0.0001			
	*d*_lim_ (µm)	0.125	<0.0001	0.994		0.050
12	*k* (µm^(*n* − 1)^min^−1^)	0.092	<0.0001		0.993	
	*n* (-)	2.11	<0.0001			

**Table 4:** Statistical summary of parameter estimation using the warped-time kinetics model.

Run	Parameter	Value	*p*-Value	R^2^	Adj. R^2^	SSR
	*d*_lim_ (µm)	0.185	0.0003	0.976		0.173
1	*k*_0_ (min^−n^)	1.34	<0.0001		0.972	
	*n* (-)	0.368	<0.0001			
	*d*_lim_ (µm)	0.166	<0.0001	0.992		0.045
2	*k*_0_ (min^−n^)	2.12	<0.0001		0.991	
	*n* (-)	0.281	<0.0001			
	*d*_lim_ (µm)	0.165	<0.0001	0.990		0.056
3	*k*_0_ (min^−n^)	2.49	<0.0001		0.989	
	*n* (-)	0.281	<0.0001			
	*d*_lim_ (µm)	0.159	<0.0001	0.996		0.020
4	*k*_0_ (min^−n^)	3.06	<0.0001		0.996	
	*n* (-)	0.231	<0.0001			
	*d*_lim_ (µm)	0.174	<0.0001	0.987		0.078
5	*k*_0_ (min^−*n*^)	1.89	<0.0001		0.985	
	*n* (-)	0.313	<0.0001			
	*d*_lim_ (µm)	0.161	<0.0001	0.996		0.020
6	*k*_0_ (min^−*n*^)	2.83	<0.0001		0.996	
	*n* (-)	0.240	<0.0001			
	*d*_lim_ (µm)	0.164	<0.0001	0.992		0.039
7	*k*_0_ (min^−*n*^)	3.09	<0.0001		0.991	
	*n* (-)	0.250	<0.0001			
	*d*_lim_ (µm)	0.157	<0.0001	0.999		0.005
8	*k*_0_ (min^−*n*^)	3.71	<0.0001		0.999	
	*n* (-)	0.203	<0.0001			
	*d*_lim_ (µm)	0.166	<0.0001	0.992		0.047
9	*k*_0_ (min^−*n*^)	2.17	<0.0001		0.991	
	*n* (-)	0.287	<0.0001			
	*d*_lim_ (µm)	0.160	<0.0001	0.995		0.025
10	*k*_0_ (min^−*n*^)	2.88	<0.0001		0.994	
	*n* (-)	0.236	<0.0001			
	*d*_lim_ (µm)	0.190	<0.0001	0.989		0.120
11	*k*_0_ (min^−*n*^)	0.948	<0.0001		0.987	
	*n* (-)	0.343	<0.0001			
	*d*_lim_ (µm)	0.174	<0.0001	0.996		0.032
12	*k*_0_ (min^−*n*^*)*	1.59	<0.0001		0.995	
	*n* (-)	0.306	<0.0001			

**Table 5:** Statistical summary of the estimated MLRM coefficients correlating the breakage rate constant *k* of the *n*th-order kinetics model with the microhydrodynamic parameters.

Approach	Best Model	Parameter			Model			
		Symbol ^a^	Coefficient ^b^	*p*-Value	R^2^	Adj. R^2^	SSR	*p*-Value
First-order MLRM	*BM*_1_	*a* (mHz)	5.66×10^−3^	3.91 × 10^−5^	0.922	0.911	0.253	3.91 × 10^−5^
	*BM*_2_	*σ*_b_^max^ (GPa)	1.52 × 10^−1^	8.45 × 10^−3^	0.978	0.970	0.073	1.12 × 10^−5^
		*a* (mHz)	4.68 × 10^−3^	4.05 × 10^−5^				
	*BM*_3_	*σ*_b_^max^ (GPa)	1.52 × 10^−1^	1.86 × 10^−2^	0.978	0.964	0.073	1.50 × 10^−4^
		*α_b_* (µm)	1.86 × 10^−3^	9.24 × 10^−1^				
		*a* (mHz)	4.64 × 10^−3^	6.88 × 10^−4^				
	*BM*_4_	*σ*_b_^max^ (GPa)	1.51 × 10^−1^	1.08 × 10^−1^	0.978	0.955	0.073	1.47 × 10^−3^
		*α*_b_ (µm)	2.07 × 10^−3^	9.41 × 10^−1^				
		*a* (mHz)	4.63 × 10^−3^	1.19 × 10^−2^				
		*Πσ*_y_ ( × 10^−16^ J^2^/m^6^s)	1.06 × 10^−4^	9.90 × 10^−1^				
Second-order MLRM	*BM*_1_	*a* (mHz)	5.66 × 10^−3^	3.91 × 10^−5^	0.922	0.911	0.253	3.91 × 10^−5^
	*BM*_2_	*σ*_b_^max^ (GPa)	1.52 × 10^−1^	8.45 × 10^−3^	0.978	0.970	0.073	1.12 × 10^−5^
		*a* (mHz)	4.68 × 10^−3^	4.05 × 10^−5^				
	*BM*_3_	*σ*_b_^max^ (GPa)	1.40 × 10^−1^	1.90 × 10^−2^	0.982	0.971	0.060	9.14 × 10^−5^
		*a* (mHz)	6.11 × 10^−3^	7.74 × 10^−3^				
		*a*^2^ (mHz^2^)	−7.38 × 10^−6^	3.39 × 10^−1^				
	*BM*_4_	*a* (mHz)	1.22 × 10^−2^	1.27 × 10^−3^	0.994	0.988	0.020	1.14 × 10^−4^
		*Πσ*_y_ (×10^−16^ J^2^/m^6^s)	1.19 × 10^−2^	1.81 × 10^−2^				
		*α*_b_^2^ (µm^2^)	−1.16 × 10^−2^	4.14 × 10^−2^				
		*a*^2^ (mHz^2^)	−3.64 × 10^−5^	4.88 × 10^−3^				
MLRM with interaction terms	*BM*_1_	*a* (mHz)	5.66 × 10^−3^	3.91 × 10^−5^	0.922	0.911	0.253	3.91 × 10^−5^
	*BM*_2_	*σ*_b_^max^ (GPa)	1.52 × 10^−1^	8.45 × 10^−3^	0.978	0.970	0.073	1.12 × 10^−5^
		*a* (mHz)	4.68 × 10^−3^	4.05 × 10^−5^				
	*BM*_3_	*a* (mHz)	1.87 × 10^−2^	2.74 × 10^−4^	0.992	0.988	0.024	9.98 × 10^−6^
		*α*_b_*a* (µm.mHz)	−3.25 × 10^−3^	1.20 × 10^−3^				
		*aΠσ*_y_ (×10^−16^ mHz J^2^/m^6^s)	−9.77 × 10^−5^	6.67 × 10^−3^				
	*BM*_4_	*a* (mHz)	1.53 × 10^−2^	3.20 × 10^−4^	0.998	0.997	0.005	6.50 × 10^−6^
		*Πσ*_y_ (×10^−16^ J^2^/m^6^s)	1.86 × 10^−2^	1.54 × 10^−2^				
		*α*_b_*a* (µm.mHz)	−2.48 × 10^−3^	1.31 × 10^−3^				
		*aΠσ*_y_ (×10^−16^ mHz J^2^/m^6^s^2^)	−1.51 × 10^−4^	8.96 × 10^−4^				

^a^: Statistically insignificant (*p*-value > 0.01) parameters are shown in bold. *Πσ*_y_ is treated as a single parameter, as *σ*_y_ is a constant. ^b^: The coefficients have the units that make the MLRM equation dimensionally homogeneous.

**Table 6:** Statistical summary of the estimated MLRM coefficients correlating the breakage rate constant *k* of the *n*th-order kinetics model with the process parameters/bead properties (the purely empirical model).

Approach	Best Model	Parameter			Model			
		Symbol ^a^	Coefficient ^b^	*p*-Value	R^2^	Adj. R^2^	SSR	*p* Value
First-order MLRM	*BM*_1_	*c* (-)	1.31	1.51 × 10^−3^	0.783	0.752	0.707	1.51 × 10^−3^
	*BM*_2_	*c* (-)	1.08	2.71 × 10^−2^	0.809	0.745	0.623	6.96 × 10^−3^
		*Y*_b_ (GPa)	4.02 × 10^−3^	4.03 × 10^−1^				
	*BM*_3_	*c* (-)	3.68	2.43 × 10^−2^	0.908	0.853	0.299	5.02 × 10^−3^
		*ρ*_b_ (kg/m^3^)	−1.17 × 10^−3^	6.76 × 10^−2^				
		*Y*_b_ (GPa)	3.07 × 10^−2^	6.17 × 10^−2^				
	*BM*_4_	*ω* (rpm)	3.31 × 10^−4^	3.03 × 10^−2^	0.975	0.950	0.081	1.81 × 10^−3^
		*c* (-)	3.68	5.37 × 10^−3^				
		*ρ*_b_ (kg/m^3^)	−2.33 × 10^−3^	7.04 × 10^−3^				
		*Y*_b_ (GPa)	5.95 × 10^−2^	6.62 × 10^−3^				
Second-order MLRM	*BM*_1_	*c*^2^ (-)	2.98	4.78 × 10^−4^	0.843	0.820	0.512	4.78 × 10^−4^
	*BM*_2_	*c*^2^ (-)	2.52	8.13 × 10^−3^	0.868	0.824	0.431	2.30 × 10^−3^
		*Y*_b_^2^ (GPa^2^)	4.81 × 10^−6^	3.27 × 10^−1^				
	*BM*_3_	*c* (-)	−4.23	5.86 × 10^−2^	0.930	0.888	0.228	2.57 × 10^−3^
		*ω*^2^ (rpm^2^)	4.72 × 10^−8^	8.03 × 10^−2^				
		*c*^2^ (-)	9.30	2.45 × 10^−2^				
	*BM*_4_	*ω* (rpm)	3.31 × 10^−4^	3.03 × 10^−2^	0.975	0.950	0.081	1.81 × 10^−3^
		*c* (-)	3.68	5.37 × 10^−3^				
		*Y*_b_ (GPa)	−1.56	7.06 × 10^−3^				
		*Y*_b_^2^ (GPa^2^)	7.77 × 10^−3^	7.04 × 10^−3^				
MLRM with interaction terms	*BM*_1_	*ωc* (rpm)	3.83 × 10^−4^	5.38 × 10^−4^	0.838	0.814	0.530	5.38 × 10^−4^
	*BM*_2_	*ω* (rpm)	−3.01 × 10^−4^	4.44 × 10^−2^	0.922	0.895	0.256	4.83 × 10^−4^
		*ωc* (rpm)	1.07 × 10^−3^	8.10 × 10^−3^				
	*BM*_3_	*Y*_b_ (GPa)	−8.32 × 10^−1^	2.44 × 10^−3^	0.980	0.969	0.063	1.07 × 10^−4^
		*ωc* (rpm)	9.56 × 10^−4^	4.51 × 10^−4^				
		*ρ*_b_*Y*_b_ (GPa.kg/m^3^)	1.38 × 10^−4^	2.43 × 10^−3^				
	*BM*_4_	*ω* (rpm)	−3.01 × 10^−4^	2.49 × 10^−3^	0.993	0.985	0.024	1.61 × 10^−4^
		*ωc* (rpm)	1.50 × 10^−3^	7.58 × 10^−4^				
		*cρ*_b_ (kg/m^3^)	−1.87 × 10^−3^	1.42 × 10^−2^				
		*cY*_b_ (GPa)	5.02 × 10^−2^	1.17 × 10^−2^				

^a^: Statistically insignificant (*p*-value > 0.05) parameters are shown in bold. ^b^: The coefficients have the units that make the MLRM equation dimensionally homogeneous.

**Table 7:** Statistical summary of the parameters of the *n*th-order model fitting vs. predictions by the kinetic–microhydrodynamic model (Equation (17)) and the purely empirical model (Equation (18)).

Run	Direct Fit, Prediction	*d*_lim_ (µm)	*k* (µm*^n^*^−1^min^−1^)	*n* (-)	R^2^	SSR
9	*n*th order model fit	0.142	0.264	2.00	0.996	0.023
	Prediction by Equation (17)	0.156	0.280	1.92	0.995	0.028
	Prediction by Equation (18)	0.156	0.383	1.92	0.986	0.086
10	*n*th order model fit	0.136	0.527	2.12	0.996	0.023
	Prediction by Equation (17)	0.139	0.574	2.09	0.995	0.026
	Prediction by Equation (18)	0.139	0.676	2.09	0.837	0.257
11	*n*th order model fit	0.093	0.023	2.09	0.999	0.015
	Prediction by Equation (17)	0.156	0.041	1.92	0.978	0.156
	Prediction by Equation (18)	0.156	−0.185	1.92	N/A	N/A
12	*n*th order model fit	0.125	0.092	2.11	0.994	0.050
	Prediction by Equation (17)	0.139	0.093	2.09	0.993	0.055
	Prediction by Equation (18)	0.139	0.021	2.09	0.644	2.65

## Data Availability

Data are contained within the article and Supplementary Materials.

## Supplementary Materials

The following are available online at https://www.mdpi.com/article/10.3390/pharmaceutics13071055/s1: Table S1: Average stirrer power per unit volume *P*_w_, apparent shear viscosity *µ*_L_, and density *ρ*_L_ of the milled drug suspensions, as well as the calculated microhydrodynamic parameters for the wet milling experiments; Table S2: Statistics of the estimated MLRM coefficients, including the intercept, correlating the breakage rate constant *k* of the *n*th-order kinetics model to the microhydrodynamic parameters; S1: Sample MATLAB code for the microhydrodynamic calculations; S2: R code for the subset selection algorithm.

## Abbreviations

Symbols used	
*a*	Average frequency of drug particle compressions, Hz
*c*	Bead loading or fractional volumetric concentration of the beads, –
*d*	Particle diameter (size), m
*e*	Restitution coefficient, –
*F*_b_^n^	Average maximum normal force during collision of two identical elastic beads, N
*g*_0_	Radial distribution function, –
*i*	Observation (run) index, –
*I*	Maximum # of observations, –
*j*	Predictor index, –
*J*	Maximum # of allowed predictors, –
*k*	Breakage rate constant in Equations.(2) and (3), min^–1^ and µm^1–n^∙min^–1^, respectively
*k*(*t*)	Time-dependent breakage rate parameter in Equation (4), min^–1^
*k*_0_	Breakage rate constant in Equation (6), min^–n^
*K*	Coefficient obtained from an empirical correlation, –
*n*	Exponent in the kinetic models, –
*p*	Probability for a single drug particle to be caught between the beads, –
PSD	Particle size distribution
*P*_w_	Average stirrer power per unit volume, W/m^3^
*Q*	Volumetric flow rate, m^3^/s
*R*	Radius, m
*R*_diss_	Dissipation coefficient of the bead, –
*R*_diss0_	Dissipation coefficient when relative motion of the bead/liquid is absent, –
*t*	Milling time, s
*T*	Available # of predictors for a given MLRM approach, –
*V*	Volume, m^3^
*u*_b_	Average bead oscillation velocity, m/s
*V*_m_	Volume of the milling chamber, m^3^
*Y*	Young’s modulus, Pa
*Y**	Reduced elastic modulus for the bead–drug contact, Pa
Greek letters	
*α*_b_	Radius of the contact circle formed at the contact of two beads, m
*ε*_coll_	Energy dissipation rate due to partially inelastic bead–bead collisions, W/m^3^
*ε*_ht_	Power spent on shear of milled suspension of the slurry at the same shear rate, but calculated (measured) when no beads were present in the flow, W/m^3^
*ε*_m_	Non-dimensional bead–bead gap thickness at which the lubrication force stops increasing and becomes a constant, –
*ε*_tot_	Total energy dissipation rate, W/m^3^
*ε*_visc_	Energy dissipation rate due to both the liquid–beads viscous friction and lubrication, W/m^3^
Φ	Warped time, min^n^
*η*	Poisson’s ratio, –
*θ*	Granular temperature, m^2^/s^2^
*µ*_L_	Apparent shear viscosity, Pa·s
*ν*	Frequency of single-bead oscillations, Hz
*Π*	Energy dissipation rate attributed to the deformation of drug particles per unit volume, W/m^3^
*Πσ*_y_	Pseudo energy dissipation rate, J^2^/m^6^s
*ρ*	Density, kg/m^3^
*σ*_b_^max^	Maximum bead contact pressure at the center of the contact circle, Pa
*σ*_y_	Contact pressure in drug particle when the fully plastic condition is obtained, Pa
Mean residence time, s	
*ω*	Stirrer (rotational) speed, rpm
*Ψ*	Volumetric fraction of drug particles in the drug suspension, –
Indices	
b	Bead
cm	Chamber of the mill
L	Equivalent liquid (milled drug suspension)
m	Mill
*p*	Drug particle
sm	Suspension in the mill
T	Holding tank
50	Median (50% passing) particle size
90	90% passing particle size

## Appendix A

The mathematical expressions for the effective drag (dissipation) coefficient *R*_diss_ and the microhydrodynamic parameters can be found in the work of Eskin et al. [49]. They are reported here for the sake of completeness. Wylie et al. [71] give *R*_diss_ as:

(A1) $$R_{\mathrm{diss}}=R_{\mathrm{diss}\;0}\left(c\right)+K\left(c\right)d_{b}\rho_{L}\theta^{1/2}/\mu_{L}$$

where *ρ*_L_ is the density of the suspension and *K* is a coefficient given by an empirical correlation of bead concentration *c*:

(A2) $$K\left(c\right)=\frac{\left(0.096+0.142c^{0.212}\right)}{{\left(1-c\right)}^{4.454}}$$

*R*_diss0_ in Equation (A1) is the dissipation coefficient taking into account the squeezing of the milled suspension film between two approaching beads, and is expressed as follows:

(A3) $$R_{\mathrm{diss}\;0}\left(c\right)=k_{1}\left(c\right)-k_{2}\left(c\right)\ln\varepsilon_{m}$$

In Equation (A3), *ε*_m_ is the non-dimensional bead–bead gap thickness at which the lubrication force stops increasing and becomes a constant. According to Sangani et al. [72], *ε*_m_ can be taken as 0.003. *k*_1_ and *k*_2_ in Equation (A3) are computed using the following equation:

(A4) $$k_{1}\left(c\right)=1+3\sqrt{c/2}+\left(135/64\right)c\ln c+11.26c\left(1-5.1c+16.57c^{2}-21.77c^{3}\right)$$

(A5) $$k_{2}\left(c\right)=cg_{0}$$

Equations (A1)–(A5) were used in Equation (8) of the main text to calculate the granular temperature *θ*. With the known *θ* value, all other microhydrodynamic parameters were calculated, as discussed in the main text and below, using the expressions derived by Eskin et al. [49]. The average maximum normal force *F*_b_^n^ during the collision of two identical elastic beads was calculated using the following equation:

(A6) $$F_{b}^{n}=1.96{\left(\frac{Y_{b}}{1-\eta_{b}^{2}}\right)}^{2/5}\rho_{b}^{3/5}R_{b}^{2}\theta^{3/5}$$

where *Y*_b_ and *ɳ*_b_ are Young’s modulus and Poisson’s ratio of the bead material, *ρ*_b_ is the bead density, and *R*_b_ is the bead radius. The probability *p* of a single drug particle with radius *R*_p_ being caught between beads was estimated as the ratio of the volume containing the caught particles to the volume of milled drug suspension falling on a pair of the milling beads, and is expressed as:

(A7) $$p=0.97\frac{c}{1-c}{\left[\frac{\rho_{b}\left(1-\eta_{b}^{2}\right)}{Y_{b}}\right]}^{2/5}\theta^{2/5}\frac{R_{p}}{R_{b}}$$

Finally, the reduced elastic modulus of the bead–particle contact Y* was calculated using the following equation:

(A8) $$\frac{1}{Y^{*}}=\frac{1-\eta_{b}^{2}}{Y_{b}}+\frac{1-\eta_{p}^{2}}{Y_{p}}$$
