# Supplementary material for: Kinetic and Microhydrodynamic Modeling of Fenofibrate Nanosuspension Production in a Wet Stirred Media Mill
Source: Pharmaceutics. 2021 Jul 10;13(7):1055. doi: 10.3390/pharmaceutics13071055 (PMC8309173; doi:10.3390/pharmaceutics13071055)
Supplement: Supplementary file 1 [file pharmaceutics-13-01055-s001.zip › pharmaceutics-1263560-supplementary.pdf]

pharmaceutics

MDPI

Supplementary Materials: Kinetic and Microhydrodynamic Modeling of Fenofibrate Nanosuspension Production in a Wet Stirred Media Mill

Gulenay Guner, Dogacan Yilmaz and Ecevit Bilgili

Table S1. Average stirrer power per unit volume P_w, apparent shear viscosity μ_L and density ρ_L of the milled drug suspensions as well as the calculated microhydrodynamic parameters for the wet milling experiments.

Run no.	P_w (W/m³)	μ_L (mPa·s)[a]	ρ_L (kg/m³)	θ×10³ (m²/s²)	u_b (m/s)	ν (kHz)	σ_b^{max} (GPa)	α_b (μm)	a (mHz)	$\Pi\sigma_y$ (J²/m⁶s)
1	2.81×10^5	160	1030	1.28	0.06	2.15	1.84×10^{-2}	3.82	15.9	1.90×10^{12}
2	8.65×10^5	119	1030	4.32	0.10	4.32	1.58	2.41	16.7	2.73×10^{16}
3	4.90×10^5	118	1030	0.74	0.04	5.60	1.65×10^{-2}	3.42	61.7	4.55×10^{12}
4	9.48×10^5	57.4	1030	2.35	0.08	10.9	1.40	2.13	61.5	6.05×10^{16}
5	1.03×10^6	144	1020	5.15	0.11	4.31	2.43×10^{-2}	5.04	55.5	1.16×10^{13}
6	1.49×10^6	67.4	1030	12.4	0.18	7.31	1.94	2.97	43.9	1.07×10^{17}
7	1.16×10^6	81.0	1030	2.50	0.08	10.3	2.11×10^{-2}	4.37	185	2.22×10^{13}
8	2.49×10^6	32.5	1030	9.53	0.16	22.0	1.85	2.82	217	3.73×10^{17}
9	5.31×10^5	113	1030	1.77	0.07	4.44	1.97×10^{-2}	4.07	51.2	6.17×10^{12}
10	1.45×10^6	57.6	1030	7.29	0.14	9.86	1.75	2.67	64.5	1.15×10^{17}
11	1.15×10^5	174	1030	0.78	0.04	1.15	1.67×10^{-2}	3.46	5.50	5.83×10^{11}
12	5.31×10^5	123	1030	4.08	0.10	2.88	1.56	2.38	8.57	1.48×10^{16}

[a]Taken at the shear rate γ of 1000 1/s.

Table S2. Statistics of the estimated MLRM coefficients, including the intercept, correlating the breakage rate constant k of the nth-order kinetic model to the microhydrodynamic parameters.

Fit	Parameter			Model			
	Symbol[a]	Coefficient[b]	p-Value	R^2	Adj R^2	SSR	p-Value
First-order MLRM, BM_2	**Intercept** (μm$^{(n-1)}$min^{-1})	1.44×10^{-1}	1.76×10^{-1}	0.828	0.799	0.182	1.72×10^{-3}
	a (mHz)	4.65×10^{-3}	1.72×10^{-3}				
First-order MLRM, BM_3	Intercept (μm$^{(n-1)}$min^{-1})	6.15×10^{-1}	3.65×10^{-3}	0.963	0.948	0.039	2.61×10^{-4}
	α_b (μm)	-1.44×10^{-1}	7.80×10^{-3}				
	a (mHz)	4.85×10^{-3}	1.08×10^{-4}				
First-order MLRM, BM_4	**Intercept** (μm$^{(n-1)}$min^{-1})	5.49×10^{-1}	1.96×10^{-2}	0.969	0.945	0.033	1.82×10^{-3}
	α_b (μm)	-1.23×10^{-1}	4.76×10^{-2}				
	a (mHz)	4.43×10^{-3}	2.79×10^{-3}				
	$\Pi\sigma_y$ (×10^{-16} J²/m⁶s)	3.66×10^{-3}	4.46×10^{-1}				
First-order MLRM, BM_5	**Intercept** (μm$^{(n-1)}$min^{-1})	5.43×10^{-1}	1.53×10^{-1}	0.969	0.927	0.033	1.36×10^{-2}
	σ_b^{max} (GPa)	1.37×10^{-3}	9.82×10^{-1}				
	α_b (μm)	-1.21×10^{-1}	1.72×10^{-1}				
	a (mHz)	4.44×10^{-3}	1.28×10^{-2}				
	$\Pi\sigma_y$ (×10^{-16} J²/m⁶s)	3.55×10^{-3}	6.24×10^{-1}				
Second-order MLRM, BM_4	**Intercept** (μm$^{(n-1)}$min^{-1})	5.50×10^{-1}	1.00×10^{-2}	0.975	0.956	0.026	1.16×10^{-3}
	α_b (μm)	-1.56×10^{-1}	8.26×10^{-3}				
	a (mHz)	8.05×10^{-3}	2.64×10^{-2}				
	a^2 (mHz²)	-1.35×10^{-5}	2.39×10^{-1}				
Second-order MLRM, BM_5	**Intercept** (μm$^{(n-1)}$min^{-1})	3.65×10^{-1}	1.98×10^{-2}	0.995	0.989	0.005	8.36×10^{-4}
	α_b (μm)	-1.16×10^{-1}	9.76×10^{-3}				
	a (mHz)	9.23×10^{-3}	4.92×10^{-3}				
	$\Pi\sigma_y$ (×10^{-16} J²/m⁶s)	7.85×10^{-3}	3.85×10^{-2}				

	$\boldsymbol{a^2}$ (mHz²)	-2.28×10^{-2}	2.70×10^{-2}				
MLRM with interaction terms, BM_4	**Intercept** ($\mu m^{(n-1)}min^{-1}$)	7.11×10^{-2}	1.37×10^{-1}	0.987	0.978	0.013	2.3×10^{-4}
	a (mHz)	1.63×10^{-2}	1.68×10^{-3}				
	$\alpha_b a$ (μm.mHz)	-2.81×10^{-3}	3.91×10^{-3}				
	$\boldsymbol{a\Pi\sigma_y}$ ($\times10^{-16}$ mHz J^2/m^6s^2)	-7.35×10^{-5}	2.98×10^{-2}				
MLRM with interaction terms, BM_5	**Intercept** ($\mu m^{(n-1)}min^{-1}$)	4.26×10^{-2}	6.40×10^{-2}	0.999	0.997	0.001	1.06×10^{-4}
	a (mHz)	1.25×10^{-2}	1.32×10^{-3}				
	$\boldsymbol{\sigma_b^{max} a}$ (GPa.mHz)	2.18×10^{-3}	1.33×10^{-2}				
	$\alpha_b a$ (μm.mHz)	-1.91×10^{-3}	4.16×10^{-3}				
	$a\Pi\sigma_y$ ($\times10^{-16}$mHz J^2/m^6s^2)	-1.45×10^{-4}	2.70×10^{-3}				

[a]Statistically insignificant (p-value > 0.01) parameters are **bolded**. $\Pi\sigma_y$ is treated as a single parameter as σ_y is a constant.
[b]The coefficients have the units that make the MLRM equation dimensionally homogeneous.

S1. Sample MATLAB code for the microhydrodynamic calculations

Main.m

```
global Pw         % Power applied to the stirrer per unit volume, W/(m^3)
global Db         % Milling bead diameter, m
global c          % solids volumetric concentration
global g0         % radial distribution function
global k          % restitution coefficient for bead-bead collisions
global Rdiss0 % dissipation coefficient taking into account squeezing the liquid film
global ub         % average particle oscillation velocity, m/s
global v          % frequency of the single bead oscillation, 1/s
global Em         % non-dimensional particle-particle gap thickness at which the lubrication force stops continous
increase and becomes a constant
global visliq    % dynamic viscosity of the equivalent liquid to be measured from viscometer, kg/ms
global dliq    % density of the equivalent liquid, kg/(m^3)
global dbeads    % density of the media beads, kg/(m^3)
global K             % empirical correlation coefficient for correction for large Reynolds numbers
global Eht             % granular energy balance for a well-mixed slurry in the mill
global Rb    %m radius of bead
global etab %Poisson ratio of the bead
global Yb    %Young modulus of the bead
global Rp    %radius of the particle
global eps    %volume fraction of the drug in the suspension
global Ystar %reduced elastic modulus of the beads
global Yp     %Young modulus of the particle
global etap    %Poisson ratio of the particle
global gamma
global clim    %maximum bead concentration

y0=[1e-6]; % case with 1 unknown, 1 equation, the initial guess was gotten from Eskin's paper at 3200rpm

% Solver based on medium scale Gauss-Newton optimization with quadcubic line search
% Do NOT change the solver parameters below:
options=optimset('Diagnostics','on','Display','iter','Largescale','off','TolX',[1e-6],'TolFun',[1e-10],...
'MaxIter',[2e4],'MaxFunEvals',[2e4],'TolCon',[1e-10],'DiffMaxChange',[1e-8],'DiffMinChange',[1e-10]);
[y,fval,exitflag,output]=fsolve(@Equationset,y0,options);

% y(1) is equal to the granular temperature theta
ub=sqrt((8/pi)*y(1)); %m/s
V=((24*c*g0)/Db)*sqrt(y(1)/pi); %Hz
v=V/1000 ; %KHz
```

```
Fbn=1.96*(Yb/(1-etab^2))^(2/5)*(dbeads^(3/5))*(Rb^2)*(y(1)^(3/5));
Alphab=(0.75*(1-etab^2)/Yb*Rb*Fbn)^(1/3); %m
alphab=Alphab*10^6 ; %micrometer
sigmabmax=3*Fbn/(2*pi*(Alphab)^2)/10^9; %GPa
p=0.97*c/(1-c)*((dbeads*(1-etab^2)/Yb)^(2/5))*(y(1)^(2/5))*Rp/Rb;
A=p*V; %Hz
a=A*1000; %mHz
pisigmaA=2.23*(c^2)*2*g0/((pi^(5/2))*eps);
pisigmaB=(Yb/(1-etab^2))^(18/15);
pisigmaC=((Ystar/Yp)^gamma);
pisigmaD=(dbeads^(4/5))/Rb^2*(y(1)^(13/10));
pisigma=2.23*(c^2)*2*g0/((pi^(5/2))*eps)*((Yb/(1-
etab^2))^(18/15))*((Ystar/Yp)^gamma)*(dbeads^(4/5))*Rp/Rb^2*(y(1)^(13/10)); %(J2/m6.s)
granulartemperature=y; %m2/s2

% END OF MAIN PROGRAM %
g0
granulartemperature %m2/s2
ub %m/s
v %KHz
sigmabmax %GPa
alphab %(micrometer)
a %mHz
pisigma %J2/m6.s)
format shortg
Result=[g0; y(1); ub; v; sigmabmax; alphab; a; pisigma]
```

Equationset.m

```
global Pw        % Power applied to the stirrer per unit volume, W/(m^3)
global Db        % Milling bead diameter, m
global c         % solids volumetric concentration
global g0        % radial distribution function
global k         % restitution coefficient for bead-bead collisions
global Rdiss0 % dissipation coefficient taking into account squeezing the liquid film
global ub        % average particle oscillation velocity, m/s
global v         % frequency of the single bead oscillation, 1/s
global Em        % non-dimensional particle-particle gap thickness at which   the lubrication force stops continuous
increase and becomes a constant
global visliq    % dynamic viscosity of the equivalent liquid to be measured from viscometer, kg/ms
global dliq   % density of the equivalent liquid, kg/(m^3)
global dbeads   % density of the media beads, kg/(m^3)
global K          % empirical correlation coefficient for correction for large Reynolds numbers
global Eht          % granular energy balance for a well-mixed slurry in the mill
global Rb     %m redius pf bead
global etab   %Poisson ratio of the bead
global Yb     %Young modulus of the bead
global Rp     %radius of the particle 0min
global eps    %volume fraction of the drug in the suspension
global Ystar   %reduced elastic modulus of the beads
global Yp     %Young modulus of the particle
global etap   %Poisson ratio of the particle
```

```
global gamma
global clim %maximum bead concentration
Pw=822916.6667; % please get from data % Power applied to the stirrer per unit volume, W/(m^3)
Eht=0; % please get from data % granular energy balance for a well-mixed slurry in the mill
%other parameters
c= 0.55;% please get from data %    solids volumetric concentration i.e (50 ml bead powder *void fraction)/total volume of chamber
k= 0.76; % particle-particle restitution coef. ref. Tatsumi see coefficient of restitution paper 0.76 for YSZ, 0.9 for CPS
Em=0.003;     % non-dimesnional particle-particle gap thickness
visliq=0.048390987;%kg/(m·s) Please measure for each sample
dliq= 1026; %kg/(m^3) Please measure for each sample
dbeads=6000;%kg/(m^3)% measured
Db= 0.000405; %m please include the diameter of the bead with different sizes.
Rb= Db/2; %m radius of the bead
etab=0.2; %poisson ratio of the bead    (0.2 for YSZ 0.33 for CPS)
Yb=200*10^9; %Pa Young modulus of the bead (200 GPa for YSZ, 1.5 GPa for CPS)
Rp=13.723*10^-6; %0 min (m)radius of the particle
eps=0.07444882; %volume fraction of the drug in the suspension
Ystar=((1-etab^2)/Yb+(1-etap^2)/Yp)^(-1); %reduced elastic modulus of the beads
Yp=8.93*10^9; %Pa Young modulus of the particle
etap=0.3; %Poisson ratio of the particle
gamma=1/3;
clim=0.63;
% calculate the emprical correlation coefficient
K=(0.096+0.142*c^0.212)/((1-c)^4.454);

%Calculate the radial distribution function
g0=(1-(c/clim)^(1/3))^-1

% Dissipation Coefficient
Rdiss0=1+3*((0.5*c)^0.5)+(135/64)*c*log(c)+11.26*c*(1-5.1*c+16.57*(c^2)-21.77*(c^3))-log(Em)*g0*c;
Re=Db*dliq*(y(1)^0.5)/visliq;

Rdiss=Rdiss0+Re*K;
nb=6*c/(pi*(Db^3));
epsvis= 9*pi*visliq*Db*nb*y(1)*Rdiss;
epscoll=(12/(Db*sqrt(pi)))*(1-(k^2))*(c^2)*dbeads*g0*(y(1)^1.5);
func(1)=Pw-epsvis-epscoll-Eht;
```

S2. R code for the subset selection algorithm

```
for (i in c(1:4)) {
  print(paste("running variable", i))
  bestAdjR=0
  bestRSS=Inf
  regnames=combn(colnames(data[,!(names(data) %in% c('k'))]),i)
  for (j in c(1:length(regnames[1,]))) {
    model=paste0("k~",paste("0",paste(regnames[,j],collapse="+"),sep = "+"))
    #model=paste0("k~",paste(regnames[,j],collapse="+"))
    fit=lm(as.formula(model),data = data)
    adjR=summary(fit)$adj.r.squared
    RSS=anova(fit)["Residuals", "Sum Sq"]
```

```
    R2=summary(fit)$r.squared
    Pvalue=summary(fit)$coefficients[,4]
    Pmodel=lmp(fit)
    if (adjR>=bestAdjR) {
      bestAdjR=adjR
      bestRSS=RSS
      bestModel=model
      bestR2=R2
      bestcoef=coef(fit)
      bestPvalue=Pvalue
      bestfit=fit
      bestPmodel=Pmodel
    }

  }

  print("best model")
  print("best coef")
  print(formatC(bestcoef, format="e", digits = 2))
  print("best Pvalue")
  print(formatC(bestPvalue, format="e", digits = 2))
  print("Best R2")
  print(bestR2)
  print("adj r")
  print(bestAdjR)
  print("bestRSS")
  print(bestRSS)
  print("bestPmodel")
  print(formatC(bestPmodel, format="e", digits = 2))

}
```
